# Balancing the Scales: The Dual Role of Interleukins in Bone Metastatic Microenvironments

**DOI:** 10.3390/ijms25158163

**Published:** 2024-07-26

**Authors:** Ahmad Dawalibi, Amal Ahmed Alosaimi, Khalid S. Mohammad

**Affiliations:** 1 Department of Anatomy, College of Medicine, Alfaisal University, Riyadh 11533, Saudi Arabia; adawalibi@alfaisal.edu; 2 College of Medicine, Imam Mohammad Ibn Saud Islamic University, Riyadh 11432, Saudi Arabia; 441020691@sm.imamu.edu.sa

**Keywords:** interleukins, bone metastasis, pro-inflammatory, anti-inflammatory, tumor microenvironment, therapy

## Abstract

Bone metastases, a common and debilitating consequence of advanced cancers, involve a complex interplay between malignant cells and the bone microenvironment. Central to this interaction are interleukins (ILs), a group of cytokines with critical roles in immune modulation and inflammation. This review explores the dualistic nature of pro-inflammatory and anti-inflammatory interleukins in bone metastases, emphasizing their molecular mechanisms, pathological impacts, and therapeutic potential. Pro-inflammatory interleukins, such as IL-1, IL-6, and IL-8, have been identified as key drivers in promoting osteoclastogenesis, tumor proliferation, and angiogenesis. These cytokines create a favorable environment for cancer cell survival and bone degradation, contributing to the progression of metastatic lesions. Conversely, anti-inflammatory interleukins, including IL-4, IL-10, and IL-13, exhibit protective roles by modulating immune responses and inhibiting osteoclast activity. Understanding these opposing effects is crucial for developing targeted therapies aimed at disrupting the pathological processes in bone metastases. Key signaling pathways, including NF-κB, JAK/STAT, and MAPK, mediate the actions of these interleukins, influencing tumor cell survival, immune cell recruitment, and bone remodeling. Targeting these pathways presents promising therapeutic avenues. Current treatment strategies, such as the use of denosumab, tocilizumab, and emerging agents like bimekizumab and ANV419, highlight the potential of interleukin-targeted therapies in mitigating bone metastases. However, challenges such as therapeutic resistance, side effects, and long-term efficacy remain significant hurdles. This review also addresses the potential of interleukins as diagnostic and prognostic biomarkers, offering insights into patient stratification and personalized treatment approaches. Interleukins have multifaceted roles that depend on the context, including the environment, cell types, and cellular interactions. Despite substantial progress, gaps in research persist, particularly regarding the precise mechanisms by which interleukins influence the bone metastatic niche and their broader clinical implications. While not exhaustive, this overview underscores the critical roles of interleukins in bone metastases and highlights the need for continued research to fully elucidate their complex interactions and therapeutic potential. Addressing these gaps will be essential for advancing our understanding and treatment of bone metastases in cancer patients.

## 1. Introduction

The bone framework is the most common tissue afflicted by metastatic cancer, and it is also the site of disease with the highest morbidity [1,2]. The complex relationship within the bone microenvironment significantly influences the complicated development and advancement of bone metastases, creating substantial complexities in the management of cancerous conditions [3,4]. Bone metastases make up a multifaceted process intricately involving the infiltration of malignant cells from their originating sites to the bone structure. This cascade of events leads to substantial skeletal deterioration and the emergence of severe complications for affected individuals. Various factors, such as interleukins (ILs), play a pivotal role in modulating the progression of bone metastases by orchestrating intricate interactions between malignant cells and bone cells within the localized tumor microenvironment [5]. These inflammatory agents, which encompass IL-1, IL-6, and IL-8, have been identified as key players in facilitating osteoclastogenesis, promoting tumor proliferation, and fostering angiogenesis. These actions collectively contribute to the breakdown of bone integrity and the relentless advancement of metastatic lesions [6]. Investigating the molecular mechanisms controlling the actions of ILs in relation to bone metastasis is essential to developing tailored treatment regimens and improving the prognosis of patients suffering from advancing cancers [7]. Directed endeavors aimed at targeting specific pro-inflammatory or anti-inflammatory interleukins present promising avenues for novel therapeutic modalities aimed at alleviating the deleterious impacts of bone metastases in oncological patients. These prospects underscore the fertile grounds for prospective investigations in this specialized domain [8].

### 1.1. Background on Bone Metastases

The occurrence of bone metastasis in cancer represents a pivotal milestone in the natural course of the disease, often concomitant with notable levels of morbidity and mortality. Bone metastases develop from the dissemination of malignant cells casting from the primary neoplasm to osseous tissues, where they form secondary lesions that perturb the regular dynamics and functionality of the bone [9]. Tumor cells produce hormones and growth factors such as parathyroid hormone-related peptides (PTHrP), receptor activator of nuclear factor kappa B (NFκB) ligand (RANKL), granulocyte-macrophage colony stimulatory factor (GMCSF), IL-1, IL-6, and MIP-1α, which activate various bone cells such as osteoblasts and osteoclasts and disrupt the homeostatic balance in cancers such as breast and prostate cancer. This pathophysiologic progression not only jeopardizes the structural soundness of the skeletal framework but also gives rise to complications such as bone pain, fractures, compression of the spinal cord, and hypercalcemia, collectively known as skeletal-related events (SREs), that significantly compromise the overall well-being of the individual [10,11].

The presence of bone metastases has the potential to facilitate the dissemination of neoplastic cells to distant organs, amplifying the aggressiveness of the disease and imposing constraints on the treatment modalities that can be employed [12,13]. A deep understanding of bone metastases in the context of malignancies is crucial for developing effective therapeutic strategies. Studies have demonstrated that diverse biomarkers, such as TRACP-5b, PYD, PTHrP, and the RANKL/RANK/OPG pathway, assume pivotal roles in prognosticating and overseeing bone metastases, thereby furnishing valuable insights into disease progression and treatment efficacy [14].

The interconnections within the bone microenvironment are essential for facilitating metastasis by creating a distinctive environment in which malignant cells can establish themselves and multiply [15,16]. In this specific context, the interactions between cancerous cells and bone cells are regulated by various substances, such as interleukins (ILs), which modify the communication pathways and cellular responses. Specifically, Interleukins (ILs) such as IL-6, IL-8, IL-1β, and IL-11 have been identified as important regulators in the onset and advancement of breast cancer spreading to the bones. They promote the growth of tumors and the breakdown of bone tissue [17]. These Interleukins (ILs) have an impact on the development of osteoclasts, the survival of tumor cells, and the development of novel blood vessels in the bone microenvironment. This, in turn, influences the progression of metastatic colonization [6]. Understanding the role of ILs in the bone microenvironment is crucial for understanding the complex mechanisms that drive the progression of metastasis and for developing targeted therapies that interrupt IL-guided pathways to prevent bone metastasis. Identifying precise interleukins (ILs) associated with the progression of cancer in the bone microenvironment could offer novel treatment strategies to prevent and control the spread of metastases in breast cancer and other malignant tumors [18]. The complex interaction between ILs, cancerous cells, and bone cells highlights the significance of investigating the bone microenvironment in relation to metastasis in order to discover new knowledge and potential treatments to address bone metastases. By elucidating the intricate mechanisms governing the development of bone metastases and harnessing the information yielded by biomarkers, healthcare practitioners can refine diagnostic precision, customize therapeutic interventions, and ultimately ameliorate patient outcomes in the realm of managing bone metastases originating from cancer.

### 1.2. Pathophysiology of Bone Metastases

The complex process of cancer-related bone metastasis involves a series of dynamic interactions in the bone microenvironment that alter the colonization, inactivity, and reactivation of tumor cells. Cancer metastases to bone involve complex interactions between cancer cells and the bone microenvironment, which have significant effects on clinical outcomes. Tumor cells from primary sites can spread to create more lesions in the bone through systemic circulation, which is made possible by complex interactions between cellular constituents and molecular signals. The progression of metastatic disease is influenced by various components found in the bone niche, including different types of cells such as bone cells, vascular endothelial cells, hematopoietic stem cells (HSCs), mesenchymal stromal cells (MSCs), growth factors, and cytokines [19,20].

The bone is a favored site for the spread of cancer cells, and various factors have been identified as influencing the tendency of tumor cells to metastasize to bone. Metastases in the bone can be broadly categorized as either osteoblastic, characterized by new bone formation, or osteolytic, characterized by bone destruction as seen on radiographs [21]. In osteolytic lesions, bone destruction is primarily carried out by osteoclasts activated by products of the tumor cells, rather than the tumor cells themselves [22]. Osteoblastic lesions are the results of the activation of bone-forming osteoblasts [23]. Different factors that promote osteoclast formation, dependent on RANKL (Receptor Activator of Nuclear Factor Kappa-B Ligand) or independent of RANKL, have been associated with various types of cancer. Tumor cell products typically stimulate osteoclast formation by upregulating RANKL or downregulating OPG (Osteoprotegerin) expression, thus shifting the RANKL/OPG ratio in favor of osteoclast-mediated bone resorption [24].

The main pro-inflammatory interleukins, such as IL-1 and IL-6, play a crucial role in promoting the development and activation of osteoclasts, supporting the survival of cancer cells, and initiating the growth of new blood vessels in the bone microenvironment [7]. These interleukins play an active role in the destructive process of bone degradation in metastatic tumors, providing more insight into the molecular mechanisms that drive the spread of bone metastases [25]. On the other hand, interleukins that have anti-inflammatory properties, such as IL-4 and IL-10, may provide protection by regulating immune responses and preventing bone destruction caused by osteoclasts. This could potentially affect the development and progression of bone metastases. Interleukins (ILs) have emerged as key players in influencing the metastatic milieu of tumor growth and sculpting the bone microenvironment. Studies have highlighted the role of particular ILs, such as IL-1, IL-6, and IL-11, in enhancing osteoclastogenesis, tumor growth, and bone deterioration, underscoring their potential as targets for therapeutic intervention to hinder bone metastases [26].

Understanding how interleukins regulate the balance and adjustments in the bone microenvironment is crucial for developing effective treatments that target the inflammatory processes. It is also important to utilize the potential protective effects of anti-inflammatory interleukins in managing bone metastatic conditions [6]. In addition, studying the complex signaling pathways involved in the interactions between cancerous cells and bone cells mediated by interleukins can provide valuable information about potential targets for therapy and biomarkers for predicting and monitoring the spread of cancer to the bones [27,28]. This comprehensive understanding of the role of interleukins in bone metastasis provides the basis for innovative therapeutic approaches and tailored treatments to improve clinical outcomes and enhance patients’ standard of life.

### 1.3. Introduction to Interleukins

Interleukins (ILs) are a group of cytokines that play crucial roles in the regulation of immune responses, inflammation, and hematopoiesis [29]. They are produced by various cells, including lymphocytes, macrophages, and endothelial cells, and function primarily as signaling molecules that mediate communication between cells of the immune system. These molecules can be broadly divided into two main categories: pro-inflammatory and anti-inflammatory [30]. Each category showcases unique functions in steering immune responses. Proinflammatory interleukins, such as IL-1, IL-6, IL-11, and IL-17, promote inflammation and are often involved in the initial immune response to infection or injury [31,32]. In contrast, anti-inflammatory interleukins, such as IL-10 and IL-4, serve to limit and resolve inflammatory responses, preventing excessive tissue damage and promoting healing [33,34,35]. The balance between these opposing forces is critical in various pathological conditions, including cancer [36]. Interleukin (IL) receptors play a crucial role in mediating various immune responses. The IL-1 receptor family consists of ligand-binding chains (IL-1R1, IL-1R2, IL-1R4, IL-1R5, IL-1R6), accessory chains (IL-1R3, IL-1R7), signaling inhibitors (IL-1R2, IL-1R8, IL-18BP), and orphan receptors (IL-1R9, IL-1R10) [37]. Additionally, cytokines like IL-6 and IL-11 bind to unique non-signaling α-receptors (IL-6R and IL-11R) that recruit the signal-transducing β-receptor gp130, activating intracellular signaling cascades [38]. IL-13, closely related to IL-4, utilizes the IL-4 receptor α chain (IL-4Rα) and the IL-13 receptor α1 chain for signaling specificity, with distinct JAK/STAT reactions between the two systems [39]. These receptors are essential for transmitting signals that regulate cell survival, proliferation, differentiation, and functional activity, highlighting their significance in immune responses and disease pathogenesis [40].

### 1.4. Interleukins in the Immune Modulation

Interleukins represent critical mediators in the modulation of the immune system, encompassing a myriad of functions within immune responses. These cytokines, such as IL-1, IL-6, IL-17, IL-4, IL-10, and IL-13, emerge as pivotal entities in the regulation of inflammation and the activities of immune cells [30].

Interleukin-4 (IL-4) and IL-13 are cytokines with analogous structures that play a pivotal role in the Type 2 inflammatory response orchestrated by T helper 2 (Th2) cells [41]. IL-4 is exclusive to mammals and is primarily secreted by activated CD4+ T cells, with varying amounts produced by CD4+ NK1.1+ natural killer T (NKT) cells, innate lymphoid cells (ILC2s), mast cells, basophils, eosinophils, and macrophages [42,43,44]. The Type 2 inflammatory response is initiated by large multicellular organisms like parasitic helminth worms and allergens. IL-4 exerts diverse effects on numerous target cells involved in both innate and adaptive immune responses, as well as on non-hematopoietic cells such as fibroblasts, epithelial cells, and endothelial cells [41,45]. IL-13 originates from some of the same cell sources as IL-4. Th17 cells, known for producing IL-17, contribute significantly to autoimmune diseases like RA by promoting inflammation and bone destruction, while Treg cells help maintain immune homeostasis. IL-3 is shown to inhibit the differentiation of Th17 cells and promote the development of Treg cells in an IL-2-dependent manner. IL-3 reduces the number of pathogenic Th17 cells, marked by the production of pro-inflammatory cytokines such as TNF-α and IFN-γ, and decreases osteoclastogenesis, thereby preventing bone destruction. Mechanistically, IL-3 exerts its effects by inhibiting STAT3 phosphorylation, a critical factor in Th17 cell differentiation, and enhancing STAT5 phosphorylation, which supports Treg cell development [46].

### 1.5. Influence of Interleukins on the Bone Microenvironment

**Figure 1:**
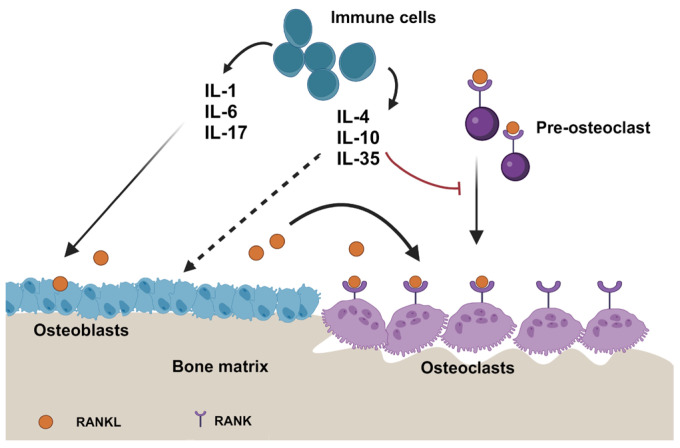
Interleukin Regulation of Bone Remodeling. This figure illustrates the complex interactions between interleukins (ILs), immune cells, osteoblasts, and osteoclasts in the bone microenvironment, highlighting the role of these cytokines in the regulation of bone remodeling and formation. Osteoblasts, depicted in blue, produce RANKL (Receptor Activator of Nuclear factor Kappa-Β Ligand), shown as orange circles. RANKL binds to its receptor RANK, expressed on pre-osteoclasts (purple cells) and mature osteoclasts (multinucleated purple cells), promoting osteoclast differentiation and activation, leading to bone resorption. Immune Cells and interleukins: Immune cells (depicted in teal) secrete various interleukins that modulate osteoclastogenesis and osteoblastogenesis. Pro-osteoclastogenic Interleukins: IL-1, IL-6, and IL-17 (indicated by solid arrows) enhance the production of RANKL by osteoblasts and directly promote the differentiation of pre-osteoclasts into mature osteoclasts. Anti-osteoclastogenic Interleukins: IL-4, IL-10, and IL-35 (indicated by red inhibitory lines) inhibit osteoclast differentiation and activity by downregulating RANKL production or directly suppressing pre-osteoclast maturation. It can also simulate osteoblast proliferation.

Understanding the complex roles of interleukins in the bone microenvironment is crucial for understanding the mechanisms underlying bone homeostasis and pathological conditions. Interleukins exhibit both pro-inflammatory and anti-inflammatory properties that significantly influence bone remodeling, immune cell interactions, and the progression of bone diseases such as osteoporosis and metastasis. Interleukins play a crucial role in the regulation of bone formation and remodeling by influencing osteoblastogenesis and osteoclastogenesis, thus maintaining bone homeostasis by acting directly on osteoclasts, osteoblasts, and osteocytes, as well as indirectly through various signaling pathways [47,48]. The production of interleukins in the bone microenvironment and the responses of bone cells to these cytokines are tightly regulated, influencing both normal bone physiology and pathological conditions like osteoporosis and inflammatory arthritis [49,50] (Figure 1).

For instance, interleukin-1α and TNF-α promote osteoclastogenesis by inducing RANKL expression, while IL-17A induces RANKL expression in osteoblasts, indirectly upregulating osteoclastogenesis [51]. Additionally, interleukin-27 has been found to inhibit RANKL-mediated osteoclastogenesis, thus ameliorating inflammatory bone destruction. Furthermore, interleukins like IL-10, IL-19, IL-4, and IL-13 have shown anti-inflammatory effects by downregulating pro-inflammatory species, contributing to the regulation of immune responses in bone tissue.

Pro-inflammatory interleukins, including IL-1 and IL-6, exhibit a propensity towards fostering osteoclastogenesis and tumor proliferation [52].

Interleukin-1 (IL-1) plays a significant role in bone physiology through its involvement in osteoclast differentiation and bone resorption. IL-1 exists in two isoforms, IL-1α and IL-1β, both of which signal through the IL-1 receptor type 1 (IL-1RI) but have different activation mechanisms. IL-1β is particularly regulated by inflammasomes, such as the NLRP3 inflammasome, which are crucial in bone and joint diseases [53]. IL-1 directly influences bone metabolism by promoting osteoclastogenesis, the process by which osteoclasts, the bone-resorbing cells, are formed. This cytokine enhances the differentiation of bone marrow-derived macrophages (BMMs) into osteoclasts through the receptor activator of NF-κB ligand (RANKL)/RANK pathway and can also drive osteoclast differentiation independently of RANKL/RANK under certain conditions, such as overexpression of IL-1RI in BMMs [54]. IL-1 activates several downstream signaling pathways, including NF-κB, JNK, p38, and ERK, which are essential for osteoclastogenesis. Additionally, IL-1 promotes the expression of microphthalmia transcription factor (MITF), which induces osteoclast-specific genes [54]. In physiological bone metabolism, IL-1 regulates osteoclast formation and activity, as evidenced by increased bone mass and decreased osteoclast numbers in IL-1α/β knockout mice [55]. Dysregulation of IL-1 signaling can lead to pathological bone conditions, such as excessive bone resorption seen in inflammatory diseases like rheumatoid arthritis [53]. Moreover, IL-1 family members are implicated in bone remodeling and skeletal homeostasis, with their dysregulation contributing to bone pathologies and metastasis in bone sarcomas [56,57]. Thus, IL-1 signaling is a complex and critical component of bone physiology, influencing both normal bone maintenance and pathological bone destruction through its regulation of osteoclast differentiation and activity.

Interleukin-6 (IL-6) plays a multifaceted role in bone physiology through several molecular pathways. Interleukin 6 (IL-6) is generated by a variety of cell types within the bone microenvironment, such as osteoblasts and osteoclasts, macrophages, T-cells, and neutrophils [58,59,60,61,62]. IL-6 is known to activate the STAT (signal transducer and activator of transcription), PI3K (phosphatidylinositol-3 kinase), and MAPK (mitogen-activated protein kinase) pathways, which are crucial for bone remodeling, inflammation, cell survival, and proliferation [63]. The binding of IL-6 to its soluble receptor (sIL-6R) is essential for the activation of the gp130/STAT3 signaling pathway, which is a critical mediator in bone homeostasis and remodeling [64]. IL-6 primarily stimulates osteoclastic bone resorption indirectly by modulating osteoblasts and the OPG/RANKL/RANK system, which coordinates the activities of osteoblasts and osteoclasts. Additionally, IL-6 enhances the expression of EP receptors and mediates prostaglandin E2 (PGE2)-induced suppression of osteoprotegerin (OPG) secretion, further influencing bone metabolism [64]. IL-6 plays a crucial role in regulating the expression of genes involved in osteoclast formation and function through various mechanisms. It has been found that IL-6 and its soluble receptor (sIL-6R) can both promote and suppress osteoclast differentiation induced by different levels of RANKL, thereby demonstrating a dual role in osteoclastogenesis [65,66]. Additionally, IL-6 produced by osteocytes under mechanical loading influences osteoblast differentiation and osteoclast activity via the JAK/STAT3 and ERK signaling pathways [67]. Furthermore, IL-6 can modulate osteocyte communication towards osteoclasts and osteoblasts, affecting bone mass by regulating gene expression involved in osteoclastogenesis and osteoblast differentiation [68]. In co-culture systems, IL-6 has been shown to induce osteoclast formation by promoting RANKL production, while in monoculture systems, it directly suppresses RANKL-induced osteoclast differentiation, with the difference attributed to the involvement of ICAM-1 from synovial cells [69] and invitro studies showed that IL-6 can act directly in osteoblasts, via both cis- and trans-signaling [70]. These cells play a crucial role in the regulation of immune responses and bone metabolism through the secretion of IL-6, which can have significant impacts on the overall health and functioning of the skeletal system. In the context of bone defect reconstruction, IL-6 has been shown to synergistically augment the osteogenic and adipogenic differentiation of human bone marrow stromal cells (hBMSCs) by promoting the translocation of BMPR1A to the cell surface, thereby amplifying BMP/Smad and p38 MAPK pathways [71]. Collectively, these pathways underscore the complex regulatory role of IL-6 in bone physiology, influencing both bone formation and resorption through a network of signaling cascades. In vitro studies showed that low concentrations of IL-17A enhance the differentiation of osteoclast precursors (OCPs) into osteoclasts in the presence of RANKL. IL-17A increases the expression of Beclin1 by inhibiting the phosphorylation of ERK and mTOR, thereby enhancing autophagy and reducing apoptosis in OCPs. This process promotes osteoclast differentiation, suggesting that IL-17A could be a potential therapeutic target for managing bone resorption in cancer patients. The findings highlight the pivotal role of the ERK/mTOR/Beclin1 pathway in this mechanism [72]. The study emphasizes the complexity of IL-17A’s effects on osteoclastogenesis and the need for further research, particularly in vivo, to fully understand its therapeutic potential [73]. IL-11, commonly classified as proinflammatory, has been documented as having the ability to extend the lifespan of osteoclast precursor cells [74]. In laboratory settings, IL-11 has been shown to promote the formation of osteoclasts by prompting osteoblast lineage cells to produce RANKL [75]. Furthermore, osteoblasts also express IL-11, and the mRNA levels in these cells experience a swift elevation when stimulated by parathyroid hormone (PTH), a substance known for its bone-strengthening properties [76,77].

This subset of interleukins incites the differentiation of osteoclasts, fortifies the viability of tumor cells, and augments angiogenesis, thus fostering the progression of bone metastases. In stark contrast, anti-inflammatory interleukins such as IL-4 and IL-10 showcase probable protective roles by orchestrating immune responses and restraining osteoclast function [52]. The modulation of the bone microenvironment, immune cell interactions, and bone remodeling processes by interleukins occurs through intricate signaling cascades like NF-κB, JAK/STAT, and MAPK [52].

Interleukin-4 (IL-4) inhibits osteoclast function through multiple molecular mechanisms that involve the modulation of key signaling pathways and transcription factors. IL-4 exerts its anti-osteoclastogenic effects primarily by blocking the receptor activator of NF-κB ligand (RANKL)-induced activation of nuclear factor activated T cells c1 (NFATc1), a master transcription factor which is essential for osteoclast differentiation, via the transcription factor STAT6 [78]. This inhibition occurs early in the signaling cascade, preventing the expression of osteoclast-specific genes such as cathepsin K, c-Fos, DC-STAMP, and TRAP [78,79]. Additionally, IL-4 suppresses the alternative NF-κB signaling pathway by increasing the expression of p105/p50 and inhibiting the translocation of p52, which is crucial for osteoclast and multinucleated giant cell formation [80]. IL-4 also influences macrophage polarization, shifting them from a pro-inflammatory M1 phenotype to an anti-inflammatory M2 phenotype, which is associated with reduced RANKL expression and subsequent osteoclastogenesis [81,82]. Moreover, IL-4 administration has been shown to reduce the infiltration of M1 macrophages and maintain M2 macrophages, thereby decreasing inflammation and osteocyte apoptosis in conditions such as steroid-induced osteonecrosis [82].

IL-10 plays a multifaceted role in bone remodeling by modulating the activities of both osteoclasts and osteoblasts. It is a potent anti-inflammatory cytokine that contributes to the maintenance of bone mass by inhibiting osteoclastic bone resorption and regulating osteoblastic bone formation [83]. Specifically, IL-10 inhibits RANK-induced osteoclastogenesis by down-regulating the expression of TREM-2, a receptor essential for effective osteoclast differentiation. This inhibition occurs through the suppression of calcium signaling pathways downstream of RANK, which are crucial for the activation of CaMK-MEK-ERK and the master regulator NFATc1 [84]. Additionally, IL-10 has been shown to negatively regulate microRNA-7025-5p (miR-7025-5p), which in turn enhances osteoblast differentiation by targeting the insulin-like growth factor 1 receptor (IGF1R) [85]. This regulatory mechanism suggests that IL-10 promotes osteoblast activity and bone formation. Furthermore, IL-10-producing osteoblasts, generated from fibroblasts through direct reprogramming, have been shown to significantly suppress osteoclast differentiation, indicating a potential therapeutic application in conditions like rheumatoid arthritis [86]. IL-10 also differentially regulates bone mesenchymal stem cells (BMSCs) in osteoclastogenesis by modulating the OPG/RANKL/RANK axis and activating the NF-κB, MAPK, and AKT pathways, which are critical for osteoclast formation and bone resorption [87]. Collectively, these findings highlight IL-10’s dual role in inhibiting osteoclast activity while promoting osteoblast differentiation.

IL-35, primarily produced by regulatory T cells, exhibits immunosuppressive properties that influence osteoclastogenesis. Studies have shown that IL-35 can inhibit the viability of osteoclast precursor cells, such as RAW264.7 cells, and promote their apoptosis in a dose-dependent manner, thereby suppressing osteoclast differentiation and activity [88]. This cytokine also downregulates the expression of key osteoclastogenic markers, including RANK and FOS, which are crucial for osteoclast differentiation and function [89]. IL-35 promotes mesenchymal stem cell (MSC) proliferation, inhibits MSC apoptosis, stimulates osteogenesis, and inhibits adipogenesis. IL-35 enhances the expression of key components of the Wnt/β-catenin pathway (β-catenin and Axin2) during MSC differentiation into osteoblasts suggesting that IL-35 regulates the balance between osteogenic and adipogenic differentiation through the Wnt/β-catenin-PPARγ signaling pathway [90].

In addition to their redundancy, these cytokines possess a multitude of functions in diverse, seemingly unrelated tissues or circumstances. They play a role in embryonic development, organ formation, differentiation, inflammatory responses, and the regeneration of various organs such as the liver, bone, cartilage, kidney, intestine, skin, and heart, as well as the central nervous and hematopoietic systems [91,92].

Overall, interleukins serve as key mediators in the complex network that governs bone metabolism and remodeling processes. Therefore, gaining a comprehensive understanding of the impact of interleukins in the bone microenvironment is essential for the development of innovative treatments that specifically target bone metastases and improve the processes involved in bone regeneration.

### 1.6. Impact on the Immune Microenvironment in Bone Metastases

Within the framework of bone metastases, the significance of interleukins in regard to the immune microenvironment is extremely important. Immune checkpoint proteins, such as PD-L1 and CTLA-4, are upregulated by cancer cells in order to prevent T-cell activation and evade immune recognition [93]. Immunosuppressive cells that impede anti-tumor immune responses, including regulatory T cells (Tregs) and myeloid-derived suppressor cells (MDSCs), are abundant in the tumor microenvironment (TME) [93]. Immunosuppressive cytokines, such as TGFβ and IL-10, are secreted by cancer cells and stromal cells [93]. These cytokines generate an immunosuppressive microenvironment that further impedes effective immune responses. Moreover, tumors’ aberrant vasculature and thick extracellular matrix serve as physical barriers prohibiting immune cells from invading and functioning [93]. Cytokines (such as IL-2 and IFN-α) can be administered to promote immune cell activation and proliferation. The immunomodulatory cytokine IL-2 is a crucial cytokine for the growth, proliferation, and differentiation of T cells. It has diverse actions including boosting NK cell activity, enhancing antibody production by B cells, and supporting regulatory T cells (Treg) [94,95]. IL-2 activates JAK-STAT, RAS-MAPK, and PI3K-AKT pathways, which are crucial for its diverse immunological effects [96,97,98,99]. IL-2 signals through receptors with varying affinities (low, intermediate, high) based on their subunit composition (IL-2Rα, IL-2Rβ, IL-2Rγ) [98]. Different immune cells express these receptors variably, influencing their sensitivity to IL-2. Immune cell infiltration and function can be enhanced by tumor microenvironment (TME) modification techniques such as reprogramming tumor-associated macrophages, targeting immunosuppressive cells, or normalizing tumor vasculature [93]. The work conducted by [99]. Cheng et al., sheds light on the extensive ramifications of the receptor activator of the nuclear factor-κB ligand (RANKL/RANK) system beyond its conventional skeletal functions, encompassing its involvement in immune modulation and the advancement of cancer. They explored the role of the RANKL/RANK system beyond its well-known function in bone metabolism. RANKL/RANK signaling is crucial in bone remodeling, primarily mediating osteoclastogenesis and bone resorption. However, it also plays significant roles in the immune system, influencing lymph node development, lymphocyte differentiation, dendritic cell survival, and T-cell activation and tolerance induction. This pathway impacts both the innate and adaptive immune responses, facilitating dendritic cell function and regulatory T cell generation, while also contributing to immune tolerance and suppression. In cancer, RANKL/RANK signaling is implicated in tumor progression and metastasis, with evidence suggesting its involvement in promoting tumor cell migration, invasion, and survival, particularly in bone metastases [99]. Furthermore, the involvement of inflammatory and immune cells within the tumor microenvironment is paramount, accentuating the regulatory role of cytokines, growth factors, and chemokines in steering the tumorigenic cascade [100]. IL-6 mediates the crosstalk that promotes the differentiation of monocyte-dendritic progenitors into immunosuppressive macrophages, which, in turn, support metastasis. This cytokine contributes to a "metastatic switch," whereby the immune environment is altered to favor tumor progression and the establishment of metastases in bone tissue. Additionally, the presence of IL-6 is linked to increased osteoclastogenesis, further aiding the breakdown of bone tissue and the establishment of bone metastases [101].

The expression of IL-1, particularly IL-1β, in bone metastases significantly influences the activation and regulation of immune cells, contributing to the complex interplay within the tumor microenvironment. IL-1β is known to drive bone metastasis by promoting tumor cell migration and invasion, as well as by facilitating the interaction between cancer cells and bone cells, which supports metastatic outgrowth [102]. In breast cancer bone metastasis, IL-1β from tumor cells and the microenvironment can inhibit primary tumor growth by impairing the infiltration of innate immune cells with potential anti-cancer functions, while simultaneously promoting enhanced tumor cell migration and osteolytic metastases [103]. This cytokine’s role in immune regulation is further highlighted by the observation that bone metastases exhibit a less active immune microenvironment compared to primary tumors, characterized by reduced tumor-infiltrating lymphocytes (TILs) and increased tumor stroma [104]. Despite this dampened immune landscape, the expression of immune checkpoint proteins such as PD-1 and PD-L1 in bone metastases suggests that immune checkpoint inhibitors could still offer therapeutic benefits for some patients [104].

IL-4 is known to induce macrophages to polarize towards an M2-like phenotype, which is typically associated with wound healing and immunosuppression, thereby promoting tumor growth and metastasis through extracellular matrix remodeling and angiogenesis [105]. Recent findings suggest that IL-4 neutralization can enhance anti-tumor immunity by reducing the generation of immunosuppressive M2 macrophages and myeloid-derived suppressor cells, while increasing tumor-specific cytotoxic T lymphocytes, thus delaying tumor progression [106]. In the context of bone metastases, immune cells, including macrophages and T lymphocytes, play dynamic roles in regulating metastatic processes such as homing, seeding, dormancy, and outgrowth [107]. Specifically, breast cancer bone metastases exhibit a less active immune microenvironment compared to primary tumors, with reduced tumor-infiltrating lymphocytes (TILs) and increased tumor stroma, which may contribute to the immune evasion observed in metastatic sites. IL-4’s role in cancer is complex; while it generally supports tumor growth, IL-4 production by cancer cells has been associated with suppressed tumor growth and loss of metastatic potential in certain models, possibly due to enhanced phagocytic behavior of tumor-associated macrophages (TAMs) [105]. These findings highlight the binary character of interleukins, which can orchestrate both pro-inflammatory and anti-inflammatory responses in immune cells, shaping the immunological landscape inside the bone microenvironment and influencing tumor growth. The detailed exploration of interleukin functions in molding immune responses and grasping the complex interplay between interleukins and immune cells in the field of bone metastasis furnishes valuable insights into possible therapeutic approaches.

## 2. Interleukins and Cancer

### 2.1. Pro-Inflammatory Interleukins and Cancer

Proinflammatory interleukins, such as interleukin-1β (IL-1β) and interleukin-18 (IL-18), play a significant role in cancer progression by modulating the tumor microenvironment and influencing various stages of tumor development. These cytokines are part of the broader inflammatory response that includes other key players like tumor necrosis factor-alpha (TNF-α) and transforming growth factor-beta (TGF-β), which collectively contribute to cancer initiation, progression, and metastasis [108].

IL1β: In a recent study, using single-cell RNA sequencing data from various breast cancer subtypes (ER+/PR+, HER2+, and TNBC), the study identified that IL1β is significantly upregulated in triple-negative breast cancer (TNBC) samples. The analysis demonstrated that plasma cells in TNBC samples interact with T cells through the intercellular adhesion molecule 2 (ICAM2)–integrin–aLb2 complex, leading to the release of IL1β. This cytokine was found to promote tumor growth by enhancing the viability and proliferation of TNBC cells (MDA-MB-231) while having a contrasting effect on non-TNBC cells (MCF-7) [109]. In pancreatic cancer, IL-6 plays a crucial role from the early stages of pancreatic intraepithelial neoplasia (PanIN) to the metastasis of pancreatic cancer cells.

IL-6: IL-6 promotes oncogenic signaling pathways and immune escape, facilitating cancer progression. IL-6 is a key biomarker for the diagnosis and prognosis of pancreatic cancer and a potential target for therapeutic interventions [110]. Neuroblastoma cells, increase IL-6 production, which promotes their survival and proliferation. The IL-6 receptor complex, consisting of IL-6Rα/gp80 and gp130, initiates signaling pathways such as Jak/STAT and Erk-1/2, which promote tumor growth and resistance to apoptosis [111].

IL-8: High levels of IL-18 are associated with worse outcomes in breast cancer patients, indicating its role in disease progression and therapy resistance [112]. Understanding the complex roles of these interleukins in cancer can lead to novel therapeutic strategies that target the inflammatory tumor microenvironment, potentially improving treatment outcomes and patient survival.

IL-17: Prostate cancer exhibits excessive expression of IL-17, which is a cytokine known for its inflammatory properties [113]. Steiner et al. conducted a comprehensive analysis of inflammatory cytokines in normal, benign hyperplastic, and malignant prostate tissues. The expression of IL-17 was infrequent in the normal prostate, but it was elevated in benign hyperplastic and malignant prostates [113]. Furthermore, a notable association was observed between the level of IL-17 and both IL-6 and IL-8 levels in malignant prostate specimens [113].

### 2.2. Pro-Inflammatory Interleukins and Bone Metastases

The complex and diverse roles that different pro-inflammatory interleukins play in the development of breast cancer bone metastases highlight how important these interleukins are in influencing the environment in which tumors grow and the course of the disease. Studies have illuminated the significant functions of key interleukins, including IL-6, IL-8, IL-1β, and IL-11, in promoting tumor growth, osteoclast development, and metastasis colonization in the skeletal system [17]. These pro-inflammatory cytokines regulate complex signaling cascades, such as NF-κB, JAK/STAT, and MAPK, that drive osteoclast production and blood vessel creation to facilitate cell migration and invasion [114]. The oncogenic attributes of these interleukins underscore their viability as plausible targets for therapeutic intervention to impede the progression of bone metastasis and amplify the effectiveness of treatments in breast cancer sufferers [100].

The complex chain of pro-inflammatory interleukins, including IL-1, IL-6, and IL-17, is an important regulator of the bone microenvironment in regard to metastasis. Pro-inflammatory ILs like IL-1, IL-6, and IL-17 have been linked to the stimulation of osteoclastogenesis and tumor proliferation, thereby influencing critical aspects of bone metastasis processes [115]. Through the activation of signaling pathways such as NF-κB, JAK/STAT, and MAPK, these cytokines significantly contribute to the progression of metastatic events [116]. These interleukins exhibit a broad range of biological functions, including supporting angiogenesis and tumor cell survival as well as osteoclast development and activation, which influences the progression of bone metastases.

IL-1: IL-1 stimulates tumor proliferation and metastasis by inducing a glycolytic state in malignant cells and releasing inflammatory molecules that aid in adipocyte lipolysis within the bone marrow [117]. A study that combines patient samples, humanized mouse models, and genetic manipulation of breast cancer cells demonstrated that β tumor-derived IL-1β significantly contributes to the metastatic process. High IL-1 β expression in tumor cells correlates with relapse in bone and other sites, indicating its potential as a biomarker. The research highlights that IL-1B enhances epithelial-to-mesenchymal transition (EMT), invasion, and migration of cancer cells, and its inhibition through IL-1 β antibodies or IL-1R antagonists like Canakinumab and Anakinra reduces metastasis. The findings underscore the therapeutic potential of targeting IL-1 β signaling to mitigate breast cancer bone metastasis [118]. IL-1β derived from the bone marrow significantly enhances the colonization of breast cancer cells in the bone. This process occurs through the activation of NFkB and CREB signaling pathways in the cancer cells, which in turn induce autocrine Wnt signaling, leading to the formation of cancer stem cell (CSC) colonies. The study demonstrates that inhibiting this pathway can prevent both CSC colony formation in the bone microenvironment and subsequent bone metastasis [119]. IL-1β has contrasting effects in primary tumors and bone metastases. In primary tumors, IL-1β inhibits growth by reducing the infiltration of anti-tumor immune cells but promotes cell migration. Conversely, in bone, IL-1β stimulates the development of osteolytic metastases. It was found that combining standard treatments (Doxorubicin and Zoledronic acid) with the IL-1 receptor antagonist Anakinra enhances therapeutic efficacy, inhibiting both primary tumor growth and metastasis. This combination therapy shifts the immune response, maintaining an anti-inflammatory signature that contrasts with standard treatments [103].

IL-6: Similarly, the involvement of IL-6 in bone metastasis is evident through its capacity to affect the settlement of tumor cells in bone via several signaling cascades, including JAK/STAT and MAPK pathways [120]. IL-6 also enhances osteoclast activity, which promotes bone breakdown and metastases. A work that investigates the role of interleukin-6 (IL-6) in cancer growth and bone metastases, concentrating on its interaction with the bone marrow microenvironment reported that bone marrow stromal cells (BMSC) generate IL-6, which activates osteoclasts and leads to bone resorption.

IL-17: The importance of IL-17 in stimulating osteoclast formation and bone degradation, propelling the development of bone metastases is well documented [114]. A study investigated the role of interleukin-17A (IL-17A) derived from lung cancer cells in osteoclastogenesis, which is crucial for bone metastasis. The study reported that inhibiting IL-17A expression in A549 lung cancer cells suppressed osteoclast formation. Conditioned medium from IL-17A-deficient A549 cells (A549-si-CM) inhibited RANKL-stimulated osteoclastogenesis and downregulated osteoclast-related genes. This inhibitory effect occurred primarily in the early stages of osteoclast differentiation by promoting apoptosis of osteoclast precursor cells. The mechanism involved increased expression of caspase-3 (CASP3), and inhibiting CASP3 reversed the anti-osteoclastogenic effect of A549-si-CM. In vivo experiments showed that mice injected with IL-17A-deficient A549 cells had reduced osteoclast activation and bone tissue destruction compared to those injected with regular A549 cells. These findings suggest that IL-17A derived from lung cancer cells promotes osteoclastogenesis by inhibiting apoptosis of osteoclast precursors and that IL-17A could be a potential therapeutic target for cancer-associated bone resorption in lung cancer patients [121].

IL-8: In a study that was looking at the role of interleukin-8 (IL-8) in prostate cancer, specifically when it has metastasized to the bones, it was found that men with prostate cancer, particularly those with bone metastases, have much greater IL-8 levels in their serum than those without bone metastases or healthy persons. Elevated IL-8 levels have been associated with the stimulation of osteoclastogenesis. This shows that IL-8 could contribute to bone resorption seen in metastatic prostate cancer. IL-8 and MCP-1 can stimulate osteoclast development in vitro, even without RANKL, which is generally required for differentiation demonstrating a RANKL-independent mechanism for osteoclastogenesis fueled by IL8 [122]. Another study demonstrates that tumor-derived IL-8 significantly stimulates osteoclast formation and bone resorption independent of the receptor activator of the nuclear factor-kappaB ligand (RANKL) pathway. IL-8 expression was notably higher in cancer cell lines with a higher osteolytic potential. The study found that conditioned media from breast cancer (MDA-MET) and lung cancer (A549) cell lines induced osteoclast formation, which was significantly inhibited by an IL-8 neutralizing antibody but not by RANK-Fc or osteoprotegerin. This indicates that IL-8 is a major contributor to tumor-induced osteolysis through mechanisms that do not involve the RANKL pathway [123].

### 2.3. Anti-Inflammatory Interleukins and Cancer

IL-10: a multifaceted anti-inflammatory cytokine, has an intricate impact on the emergence and progress of breast cancer, exhibiting both stimulating and inhibitory effects on tumor cells [124]. Multiple lines of evidence have unequivocally shown that inflammation plays a role in promoting tumor growth. Studying intratumoral chemokines offers a means to investigate the tumor microenvironment, as these molecules play a crucial role in coordinating immune cells during inflammation [125]. A recent study has identified a cluster of pro-inflammatory chemokines that are linked to a tumorigenic transgenic adenocarcinoma mouse prostate (TRAMP)-C1 cell line. Tumor-infiltrating lymphocytes (TILs) in the tumor microenvironment release a naturally occurring anti-inflammatory substance called interleukin-1 receptor antagonist (IL1RN) [125]. This substance hinders the activities of molecules that promote inflammation and is responsible for the tumor type-specific anti-inflammatory effects. As a result, tumor cells attract TILs through pro-inflammatory chemokines, leading to the creation of an anti-inflammatory environment mediated by IL1RN in the syngeneic prostate cancer model [125].

IL-4: IL-4 receptors were found to be present in lung cancer. When the IL-4 receptor-targeted agent was given, it caused significant cell death in lung cancer cells and in mice with lung cancer. The use of IL-4 cytotoxin was suggested as a new therapeutic strategy for treating lung cancer [126].

In a recent study investigating the role of interleukin-4 (IL-4) in promoting immunosuppressive myeloid cells within non-small cell lung cancer (NSCLC) lesions, in human and mouse models. It was found that IL-4 is a key factor driving the suppressive behavior of monocyte-derived macrophages within tumors. Deleting the IL-4 receptor IL-4Rα in early myeloid progenitors significantly reduced tumor burden, whereas deleting it in mature myeloid cells did not. This suggests that IL-4 acts early in the myeloid development process, likely influenced by bone marrow-derived basophils and eosinophils. Reducing basophil levels notably decreased tumor size and corrected abnormal myelopoiesis. An IL-4Rα blocking antibody, dupilumab, alongside PD-1/PD-L1 inhibitors was tested in NSCLC patients who had not responded to previous treatments. This combination led to a reduction in circulating monocytes and an increase in tumor-infiltrating CD8 T cells, with one patient showing a near-complete response after two months. These results highlight IL-4’s central role in cancer-associated immunosuppression and introduce a promising new therapy combining immune checkpoint inhibitors with cytokine-blocking strategies, suggesting a systemic approach to cancer treatment [127].

IL-30: IL-30, also known as IL-27p28 or IL-27A, is secreted by cells of myeloid origin, like macrophages, monocytes, and dendritic cells, which are the main source of IL-30. It is also produced by Kupffer cells in the liver as well as microglial cells and astrocytes [128,129,130]. Interleukin-30 (IL-30) plays a role in the interaction between prostate cancer (PC) cells and endothelial cells (ECs). It is expressed as a membrane-anchored cytokine by human PC cells and significantly promotes angiogenesis, immune modulation, and oncogenic signaling. In vitro experiments demonstrate that contact with PC cells leads to EC proliferation and increased production of angiogenic factors such as IGF1, EDN1, ANG, and CXCL10, which are further enhanced by IL-30. Additionally, IL-30 drives the phosphorylation of key signaling proteins, including Src, STAT3, STAT6, RSK1/2, c-Jun, AKT, CREB, GSK-3α/β, HSP60, and p53. Knockout of the IL-30 gene in PC cells inhibits these pro-angiogenic effects and impairs tumor angiogenesis. The study also shows that ECs, in response to IL-30 overexpression in PC cells, upregulate a wide range of immunoregulatory and cancer-driver genes, such as BCL2, CCND2, EGR3, IL6, VEGFA, KLK3, PTGS1, LGALS4, GNRH1, and SHBG [131].

### 2.4. Anti-Inflammatory Interleukins and Bone Metastases

Anti-inflammatory ILs, exemplified by IL-4, IL-10, and IL-13, demonstrate promising roles in safeguarding against bone metastasis by regulating immune reactions and impeding osteoclast function. IL-4 and IL-10 are known for their ability to inhibit the differentiation and activity of osteoclasts, thereby reducing bone resorption [81]. In the complex network of relationships found in bone metastases, anti-inflammatory interleukins, including IL-4, IL-10, and IL-13, play a key role in coordinating immune responses [41]. These well-known interleukins have a notable ability to inhibit osteoclast activity, which may have an impact on the progression of bone metastases in cancer patients. Through the suppression of inflammatory reactions and the facilitation of an immune-regulatory milieu, these anti-inflammatory interleukins could provide a counteractive force against the pro-inflammatory cues associated with tumor proliferation and bone degeneration. Several research endeavors have indicated the possible involvement of IL-4, IL-10, and IL-13 in mitigating the aggressive characteristics of cancer within bone structures, underscoring their viability as pharmacological targets for the management of bone metastases [26].

Interleukins are integral in the regulation of immune responses, particularly within the purview of bone metastases. Certain anti-inflammatory interleukins such as IL-4, IL-10, and IL-13 have been identified as possessing possible protective attributes in this context [132].

IL-4 is recognized for its anti-osteoclastic characteristics and its ability to modulate immune reactions. IL-4 dose-dependently inhibits RANKL-induced bone resorption in mature osteoclasts. IL-4 decreases TRAP expression without affecting the multinuclearity of osteoclasts and inhibits actin ring formation and osteoclast migration. This inhibition is mediated by preventing the RANKL-induced nuclear translocation of the p65 NF-κB subunit and altering intracellular Ca2+ signaling. IL-4 rapidly reduces RANKL-stimulated ionized Ca2+ levels in the blood, demonstrating its potential to counteract hypercalcemia induced by RANKL. The study also shows that mature osteoclasts from IL-4 knockout mice are more sensitive to RANKL, highlighting the crucial role of IL-4 in regulating osteoclast activity and bone resorption [133]. Osteoprotegerin (OPG) is a decoy receptor for RANKL. Both IL-4 and IL-13 were found to induce mRNA levels and protein secretion in a dose- and time-dependent manner, primarily through the activation of the STAT6 pathway. This activation is critical, as blocking STAT6 with specific inhibitors significantly decreases OPG expression. The study also shows that IL-4 and IL-13-treated HUVEC supernatants can inhibit osteoclast activity, which is partly reversed by neutralizing OPG antibodies, indicating the crucial role of OPG in this inhibitory process [134]. A recent study established a bone metastasis model of colorectal cancer (CRC) using MC-38 or CT-26 cells in mice and demonstrated that IL4Rα expression is significantly upregulated in osteoclast precursors (OCPs) stimulated by tumor-conditioned medium (CM). IL-4 signaling regulates the proliferation of OCPs via the type I IL-4 receptor, with neutrophils identified as the primary source of IL-4 in the bone marrow. IL-4 deficiency in mice inhibits OCP proliferation and subsequent osteoclastogenesis. Mechanistically, the ERK pathway is activated by IL-4/IL4R signaling, and the ERK antagonist ravoxertinib can significantly prevent bone destruction by inhibiting OCP proliferation [135]. The studies conducted in this research have successfully elucidated the contrasting impacts observed on osteoclasts, indicating a significant confirmation that the influence of IL-4 on osteoclasts is contingent upon the specific context in which it is operating. The findings presented in these studies shed light on the intricate mechanisms governing the regulation of osteoclast activity and highlight the nuanced interplay between IL-4 and osteoclast function, underscoring the need for further exploration and in-depth analysis in this area of study.

IL-10 demonstrates immunosuppressive qualities that have the potential to impede osteoblast function. To explore how osteoblasts (OSB) in the bone marrow microenvironment impact the cytotoxic activity of natural killer (NK) cells against multiple myeloma (MM) cells. A study uses the NK-92MI cell line and the MM.1S multiple myeloma cell line in various co-culture setups to assess changes in cell viability. Results indicate that the presence of OSB decreases the effectiveness of NK cells by increasing the production of IL-6 and IL-10, cytokines that impair NK cell function and promote MM cell survival. The study hypothesizes that the presence of IL-10 in the co-culture environment could be a result of a reciprocal interaction between OSB and NK cells. While OSB-induced production of IL-10 dampens NK cell activity, the precise mechanism by which OSB influences NK cells to produce IL-10 remains an area for further investigation [136].

IL-30: A study looked into the role of interleukin-30 (IL27p28) in the behavior of prostate cancer stem-like cells (PCSLCs) and its critical involvement in tumor onset and metastasis, it was found that IL30, produced by PCSLCs, significantly influences their viability, self-renewal, and tumorigenicity through autocrine and paracrine effects, primarily via the STAT1/STAT3 signaling pathway. IL30 promotes PCSLC proliferation, immune evasion, and expression of inflammatory mediators and growth factors, enhancing the metastatic potential through the CXCR5/CXCL13 and CXCR4/CXCL12 axes. Overexpression of IL30 in PCSLCs leads to increased tumor initiation, growth, and dissemination to lymph nodes and bone marrow. Conversely, silencing IL30 reduces tumorigenicity and metastasis, highlighting IL30 as a potential therapeutic target for inhibiting prostate cancer progression and recurrence [137]. In a more recent study, the same group reported that IL30 promotes PC proliferation, invasion, and migration through STAT1/STAT3 signaling and the upregulation of oncogenes, including IGF1 and CXCL5. Using CRISPR/Cas9 technology to delete IL30 in PC cells led to the suppression of these oncogenes and the upregulation of the tumor suppressor SOCS3, significantly inhibiting tumor growth and metastasis. The findings from both in vitro experiments and in vivo mouse models indicate that IL30 deletion enhances survival and reduces tumor aggressiveness, suggesting that IL30 could be a promising target for PC treatment. Clinical data analysis reveals that high IL30 expression correlates with poor prognosis, whereas high SOCS3 expression is associated with better outcomes, reinforcing the potential clinical relevance of IL30 as a therapeutic target in PC [138]. The functional profiles and regulatory capacities of anti-inflammatory interleukins in the formulation of tailored therapeutic modalities, thereby ameliorating clinical outcomes [52].

In a similar vein, IL-13 has been linked to anti-inflammatory roles, potentially aiding in ameliorating the pro-metastatic environment inherent in bone tissue [3]. The presence of these interleukins serves to counteract the prevailing pro-inflammatory milieu linked with bone tumor advancement, thereby opening up avenues for immune modulation within the bone metastatic microenvironment. These immunomodulatory agents have been identified as key players in this dynamic interaction, highlighting their significant effects on influencing the microenvironment of the bone and, in turn, the paths of tumor growth and osseous degradation. The roles of interleukins are crucial in bone metastases, influencing tumor growth, osteoclastogenesis, and metastasis through several pathways. These cytokines present promising therapeutic targets to disrupt metastatic progression and enhance patient outcomes. Targeting interleukins and their signaling pathways must be approached with extreme care. The context of IL- and the interaction with the environment has been carefully considered before, we can develop new treatments that target IL to better manage and reduce the burden of metastatic bone disease.

## 3. Signaling Pathways Involved

The involvement of interleukins in the regulation of signaling pathways related to bone metastases in cancer is crucial and complex. Various interconnected signaling cascades, such as NF-κB, JAK/STAT, and MAPK pathways, impact important cellular processes like tumor cell survival, angiogenesis, and osteoclast differentiation in the bone microenvironment [7,100,114]. These pathways act as essential mediators in the communication between tumor cells and bone cells, influencing the development of bone metastasis. The comprehension of how interleukins coordinate these signaling processes can offer valuable insights into the underlying molecular mechanisms that facilitate cancer cell colonization and bone erosion [7]. The targeting of specific pathways linked to interleukin signaling presents potential novel therapeutic avenues to inhibit tumor progression and bone deterioration [139]. Thus, by a meticulous understanding of the complex interleukin-mediated signaling in bone metastases, scientists can pinpoint crucial molecular targets for innovative treatments aimed at impeding cancer progression. The involvement of interleukins is critical in regulating the complex signaling cascades pertaining to bone metastases, notably impacting the advancement of tumors and the remodeling of bones. Among the various interleukins, those with pro-inflammatory properties, such as IL-1, IL-6, and IL-17, have emerged as significant agents fostering the generation of osteoclasts and the progression of tumors in the bone’s microenvironment [100]. These interleukins trigger vital signaling pathways like NF-κB, JAK/STAT, and MAPK, which have pivotal roles in the commencement and advancement of metastasis [117]. In contrast, interleukins with anti-inflammatory characteristics, such as IL-4, IL-10, and IL-13, demonstrate protective functions by regulating immune responses and curtailing excessive osteoclast functionality [7]. Comprehending the opposing impacts of pro-inflammatory and anti-inflammatory interleukins on signaling pathways is imperative for formulating precise therapeutic interventions aimed at managing bone metastases in breast cancer. This underscores the necessity for further exploration into their intricate interplays within the tumor-bone microenvironment [6].

The convoluted signaling pathways entailed in the anti-inflammatory responses are crucial for regulating the immune milieu in the setting of osseous metastases. Various investigations have highlighted the importance of interleukins (ILs) in governing these responses, with IL-4, IL-10, and IL-13 emerging as pivotal anti-inflammatory agents with conceivable protective roles in bone metastasis [52]. These ILs manifest regulatory impacts on immune reactions, impeding osteoclast function and potentially influencing tumor immune responses [7]. Findings have elucidated the participation of opposing regulatory mechanisms and distinct signal transduction pathways in orchestrating the anti-inflammatory effects of these ILs, providing insights into their therapeutic implications in modulating the immune-inflammatory equilibrium within the skeletal microenvironment. By delving into the signaling pathways governing the anti-inflammatory responses mediated by ILs, bespoke interventions could be formulated to exploit these molecular mechanisms for therapeutic advantages in mitigating the advancement of bone metastases. Additionally, grasping the intricate interplay between pro-inflammatory and anti-inflammatory ILs is imperative for formulating comprehensive therapeutic approaches that target the immunological landscape of the bone microenvironment, ultimately striving to enhance patient outcomes and refine the clinical handling of bone metastases within an immuno-inflammatory milieu.

### 3.1. NF-κB Pathway

Initially identified as a key player in inflammatory responses, NF-κB signaling is now understood to be involved in numerous pathways including PI3K/AKT, MAPK, JAK-STAT, and others [140]. This signaling pathway regulates crucial physiological and pathological processes such as inflammation, immune responses, and tumor microenvironment modulation. NF-κB’s role extends to a variety of human diseases, notably cancers, autoimmune and inflammatory diseases, cardiovascular conditions, metabolic disorders, neurological diseases, and even COVID-19 [141,142]. NF-κB signaling can be initiated by a wide variety of stimuli, ranging from bacterial and viral components to cytokines, growth factors, reactive oxygen species ultraviolet and ionizing radiation, and oncogenic stimuli [143]. Immune cells demonstrate distinctive and constantly changing quantitative signal patterns that activate NF-κB signaling either externally or within the cell to convey crucial biological details regarding the surrounding environment. Harmful triggers like pathogen infiltration trigger innate immune reactions and effectively encode specific information such as the amount of ligands, duration of exposure, and distance covered through the propagation of NF-κB signaling waves, resulting in the establishment of gene expression zones within responsive cells [144,145]. The key inducers of the canonical NF-κB pathway consist of TNF-α, interleukin (IL)-1β, lipopolysaccharide (LPS), and antigens. These inducers interact with receptors on the cell surface, consequently initiating NF-κB signaling activation through the involvement of multiple intermediary proteins [146,147,148,149]. The NF-κB pathway, central to signaling, is vital in cancer advancement and metastasis. It plays a crucial role in interleukin-mediated bone metastases, orchestrating pro-inflammatory responses, stimulating tumor growth, angiogenesis, and osteoclast activation in the bone microenvironment [7]. NF-κB acts as a pivotal point for transmitting signals from pro-inflammatory interleukins like IL-1 and IL-6, which promote osteoclastogenesis and cell migration and invasion [114]. Simultaneously, the NF-κB pathway interacts with critical signaling pathways, such as JAK/STAT and MAPK, fostering an environment for malignant cell survival and bone remodeling, making it a prime target for combating bone metastatic disease [150,151]. In a study that was conducted recently, it was demonstrated that the interaction between the Receptor Activator of Nuclear Factor Kappa B (RANK) and its ligand (RANKL) had a notable impact on the enhancement of migration, invasion, and metastasis of Osteosarcoma (OS) cells through the facilitation of Epithelial-Mesenchymal Transition (EMT). Significantly, it was elucidated that the RANK/RANKL axis triggers EMT by stimulating the nuclear factor-kappa B (NF-κB) pathway. Moreover, the utilization of the NF-κB inhibitor known as dimethyl fumarate (DMF) was able to effectively inhibit the migration, invasion, and EMT processes in OS cells [152]. The detailed interplay between interleukins and the NF-κB pathway provides molecular insights into the disturbed bone microenvironment and suggests possible therapeutic approaches for upsetting this intricate network to block the growth of tumors and enhance the efficacy of treatment.

### 3.2. JAK/STAT Pathway

Signal transducer and activator of transcription (STAT) and Janus kinase (JAK) signaling pathway acts as a crucial downstream mediator for a wide range of interleukins\cytokines, hormones, and growth factors. Janus kinases (JAKs) play a crucial role in regulating cellular proliferation, differentiation, and survival through various signaling pathways. JAK proteins, including JAK2 and JAK3, are involved in signal transduction cascades that impact cell functions. They interact with downstream proteins like signal transducers and activators of transcription (STATs) to modulate gene expression related to apoptosis, growth, and development [153,154]. In hematopoietic stem cells (HSCs), JAK-STAT signaling influences proliferation, survival, and self-renewal, with mutations in this pathway linked to hematologic malignancies [155]. Moreover, JAKs are implicated in tissue stress responses, where the JAK/STAT pathway interacts with c-Jun amino-terminal kinase (JNK) to balance injury-induced apoptosis and compensatory proliferation, highlighting their role in maintaining tissue homeostasis and regulating tumor growth [156].

Janus kinase 2 (JAK) binds in a non-covalent manner to cytokine receptors, causing tyrosine phosphorylation. When it is phosphorylated, a recruiting of one or more signal transducers and activators of transcription (STAT) proteins happens, which can change the activity of certain genes [157,158]. On the other hand, Protein tyrosine phosphatases may also directly dephosphorylate STAT dimers, which inhibits JAK-STAT signaling. The STAT series includes STAT1, STAT2, STAT3, STAT4, STAT5A, STAT5B, and STAT6 [159]. Every STAT protein has distinct biological functions and serves as a regulator of the survival of cells, differentiation, metabolism, and immune response. It additionally has a crucial role in malignant tumors and autoimmune illnesses. STAT1 enhances immunity against tumors, whilst STAT3 along with other proteins may cause pro-cancer inflammation [160,161]. STAT3 and NF-κB signaling have been shown to interact closely. IL-6, a gene product controlled by NF-κB signaling, is a key STAT3 activator.

Disturbances in the JAK/STAT pathway within the context of bone metastases may result in substantial consequences on the advancement of tumors and the dynamic equilibrium within the bone microenvironment, potentially affecting the efficacy of treatments [162]. Several investigations have proposed that irregular signaling in the JAK/STAT pathway can enhance various metastatic mechanisms, such as the generation of osteoclasts and the persistence of tumor cells within the bone microenvironment [162]. Interleukin-6 (IL-6) is identified as a pleiotropic cytokine that significantly influences immune regulation and tumorigenesis by activating the JAK2/STAT3 pathway. This signaling cascade is pivotal in cell proliferation, differentiation, and the formation of a tumor-promoting inflammatory microenvironment. This pathway’s abnormal activation is reported in several cancer types, including liver, breast, colorectal, gastric, lung, pancreatic, and ovarian cancers. Elevated levels of IL-6, phosphorylated JAK2, and phosphorylated STAT3 are consistently observed across these malignancies, contributing to tumor growth, metastasis, and resistance to apoptosis [163]. The strategic targeting of this pathway emerges as a promising avenue for therapeutic interventions aimed at disrupting the vicious cycles of bone metastases by thwarting pivotal molecular interactions facilitating the proliferation of cancerous cells and the degradation of bone tissues. Delving deeper into the intricate ways through which the JAK/STAT pathway impacts bone metastases could unearth innovative targets for therapy and enhance therapeutic strategies designed to combat this multifaceted metastatic process [164].

### 3.3. MAPK Pathway

This system is made up of three important enzymes: mitogen-activated protein kinase kinase kinase (MAPKKK), mitogen-activated protein kinase kinase (MAPKK), and mitogen-activated protein kinase. MAPK is responsible for phosphorylating target proteins in the cytoplasm and nucleus [140]. Mitogen-activated protein kinase (MAPK) is a member of the serine/threonine kinase family that has an important role in a variety of cellular programs, including growth, differentiation, development, transformation, inflammatory responses, and apoptosis, by transmitting, amplifying, and integrating signals from a wide range of stimuli. MAPK signaling is a unique enzymatic cascade that facilitates signal transduction from the cell surface to the nucleus via phosphorylation [140].

The signaling cascade known as the Mitogen-Activated Protein Kinase (MAPK) pathway, essential for cellular regulation and maintenance, holds great significance regarding bone metastases. Activation of this pathway significantly by IL-6 contributes to cell migration and invasion [114]. It has been demonstrated through research that adjusting the MAPK pathway can have a notable impact on the survival and growth of tumor cells, as well as their interactions within the bone microenvironment, affecting the formation and progression of bone metastases [164]. When considering interleukin-mediated bone metastases, the MAPK pathway may act as a crucial downstream signaling route, enabling pro-inflammatory interleukins like IL-6 and IL-1 [165]. This ultimately can influence the behavior of tumor cells and processes related to bone restructuring. Gaining a comprehensive understanding of how interleukins and the MAPK pathway interact in the context of bone metastases can reveal potential targets for therapeutic interventions to hinder the advancement of cancer within the bone microenvironment. Further exploration into the molecular mechanisms through which interleukins activate the MAPK pathway could lead to promising prospects for creating precise strategies to combat bone metastases effectively [164].

## 4. Therapeutic Implications of Targeting Interleukins

### 4.1. Current Therapeutic Approaches for Bone Metastases

Current therapeutic approaches for bone metastases focus on alleviating pain, preventing skeletal-related events (SREs), and improving the patient’s quality of life. The primary strategies include systemic therapies, such as chemotherapy, hormonal therapy, and targeted therapies, which address the underlying cancer. Bisphosphonates and Denosumab, are commonly used to inhibit bone resorption and reduce the risk of fractures and other SREs. Radiation therapy is employed to relieve pain and control local tumor growth, while surgery may be necessary for stabilizing fractures or decompressing the spinal cord. While traditional palliative treatments are frequently used, there is a growing trend towards utilizing more sophisticated methods like stereotactic body radiotherapy (SBRT) to enhance long-lasting results in patients with oligometastatic conditions [166]. Emerging treatments like radiopharmaceuticals, which deliver targeted radiation to bone metastases, and novel agents targeting specific molecular pathways, are showing promise in clinical trials. A multidisciplinary approach, involving oncologists, radiologists, surgeons, and palliative care specialists, is essential to tailor the treatment plan to the individual patient’s needs and disease characteristics.

### 4.2. Overview of Drugs Targeting Interleukins

**Figure 2:**
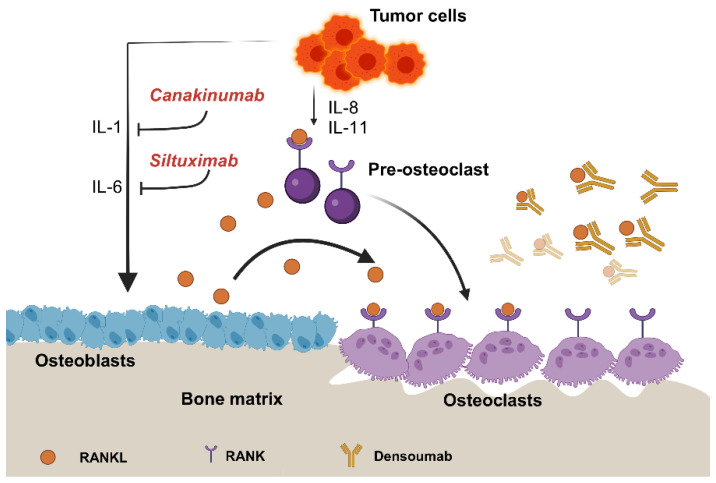
Interleukin Inhibitors and Denosumab in the Regulation of Osteoclastogenesis in Bone Metastases. This schematic illustrates the role of interleukins (IL) in the regulation of osteoclastogenesis and the therapeutic interventions targeting these pathways to manage bone metastases. Tumor cells in the bone microenvironment secrete IL-8 and IL-11, which stimulate pre-osteoclasts, promoting their differentiation into mature osteoclasts. IL-1 and IL-6 secreted by the tumor cells stimulate osteoblasts to secrete RANKL that bind to the RANK receptors on osteoclast to stimulate their activity. Osteoclasts contribute to bone resorption, which facilitates tumor growth and metastasis. The figure highlights the therapeutic targets and inhibitors involved in this process: canakinumab—An IL-1 inhibitor that blocks the signaling of IL-1, thereby reducing its stimulatory effect on osteoclastogenesis. Siltuximab—An IL-6 inhibitor that blocks IL-6, another cytokine involved in the promotion of osteoclast differentiation and activation. Both inhibitors work by preventing the activation of osteoclasts, thereby mitigating bone resorption and tumor progression. Additionally, the figure depicts the role of Denosumab, a monoclonal antibody that inhibits RANKL (Receptor Activator of Nuclear Factor Kappa-Β Ligand). RANKL is essential for the formation, function, and survival of osteoclasts. By binding to RANKL, Denosumab prevents it from interacting with its receptor RANK on pre-osteoclasts and osteoclasts, thereby inhibiting osteoclastogenesis and reducing bone resorption. This integrative approach of using interleukin inhibitors along with Denosumab offers a promising therapeutic strategy to manage bone metastases by targeting both the cytokine signaling pathways and the direct inhibition of osteoclast formation and activity.

Interleukins are pivotal in regulating immune responses and inflammation, making them prime targets for therapeutic intervention in various autoimmune diseases and cancers. The development of drugs that specifically inhibit interleukins has revolutionized treatment strategies, offering significant clinical benefits. This section provides an overview of several key interleukin-targeting drugs, including Tocilizumab, Siltuximab, Bimekizumab, ANV419, Canakinumab, and Denosumab (Table 1). These agents, through their unique mechanisms of action, demonstrate the potential to modulate immune responses, reduce inflammation, and inhibit tumor progression (Figure 2). From monoclonal antibodies to fusion proteins, these therapies represent the forefront of personalized medicine, aiming to improve patient outcomes across a spectrum of debilitating conditions.

#### 4.2.1. Tocilizumab

Tocilizumab (TCZ) is a genetically engineered humanized monoclonal antibody of the immunoglobulin G1k subclass that targets both soluble and membrane-bound interleukin 6 receptors (IL-6R) [167]. Initially approved in 2005, in Japan, for the management of Castleman’s disease, a rare condition characterized by the proliferation of plasma cells, TCZ is now authorized for the treatment of adult patients with moderate to severely active rheumatoid arthritis (RA) either as a standalone therapy or in combination with disease-modifying anti-rheumatic drugs [168]. Additionally, studies have examined its effectiveness in addressing different disorders like Crohn’s disease, systemic lupus erythematosus, Takayasu arteritis (TA), giant cell arteritis (GCA), polymyalgia rheumatica, and refractory adult-onset Still disease. Tocilizumab has been investigated for its role in reducing bone metastases by inhibiting the pro-tumor effects of IL-6. Preclinical studies have shown its potential in suppressing tumor growth and bone degradation [6]. In an Invitro study, MDA-MB-231 breast cancer cells were treated with or without anti-human IL-6 receptor (IL-6R) monoclonal antibody, and cell survival was assessed. The expressions of Stat3, VEGF, and RANK were analyzed using SDS-PAGE and immunoblotting. MDA-231 cells were implanted into mice’s left ventricles, and either anti-human IL-6R monoclonal antibodies or saline were given intraperitoneally for 28 days and the incidence of bone metastases in the hind limbs was assessed using radiography and histology. The anti-human IL-6R monoclonal antibodies reduced bone metastases in an animal model injected with MDA-231 cells, according to radiographic and histomorphometric assessments [169]. The mechanism of bone metastases inhibition involved inhibited cell survival and reducing phospho-Stat3, VEGF, and RANK expressions in MDA-231 cells [169].

#### 4.2.2. Siltuximab (CNTO 328)

It is an antibody-drug combination that specifically targets the cytokine interleukin-6 (IL-6) and shows great potential. It is highly binding to IL-6, leading to the neutralization of IL-6 bioactivity and the induction of tumor cell death [170]. Ovarian cancer has pre-clinical data suggesting that IL-6 promotes the survival of tumor cells and strengthens their resistance to chemotherapy through JAK/STAT signaling. The study found that the use of an anti-IL-6 antibody suppresses the production of cytokines, angiogenesis, and the infiltration of macrophages. This suggests that IL-6 might be a potential target for treatment in women with advanced ovarian cancer [171]. Previous studies have demonstrated that Siltuximab treatment leads to an increase in the activity of cytochrome P450 and extends the duration of disease stability. Additionally, there is a notable reduction in levels of TNF-α, IL-1, CCL2, CXCL12, and VEGF [170]. Another study has demonstrated that Siltuximab can effectively inhibit the proliferation of prostate cancer cells in vitro and enhance survival rates by decreasing cachexia levels in an animal model of prostate cancer. Furthermore, studies conducted on mice have demonstrated that Siltuximab effectively inhibits the transformation of androgen-dependent prostate cancer into a more aggressive form. Moreover, the administration of Siltuximab resulted in a reduction in blood CRP levels, which was associated with a favorable outcome in patients with treatment-resistant prostate cancer [63].

#### 4.2.3. Bimekizumab

Is a monoclonal IgG1 antibody targeting interleukin (IL)-17F and IL-17A. In a very recent two multicenter phase 3 clinical trials, BE HEARD I and II, designed to evaluate the efficacy and safety of Bimekizumab, in patients with moderate-to-severe hidradenitis suppurativa. These double-blind, randomized, placebo-controlled trials involved patients aged 18 or older, who were assigned to different treatment regimens. The primary outcome measured was the HiSCR50 response, defined as at least a 50% reduction in abscess and inflammatory nodule count without an increase in abscess or draining tunnel count, assessed at week 16. The findings showed that the HiSCR50 response was significantly higher in the Bimekizumab-treated groups compared to placebo, and the safety profiles were favorable. The data suggest that Bimekizumab offers a substantial clinical benefit and is well-tolerated in this patient population, supporting its use for managing moderate-to-severe hidradenitis suppurativa [172]. Using a human periosteum-derived cell model, it was found that both IL-17A and IL-17F promote osteogenic differentiation and bone formation. Blocking these cytokines with Bimekizumab, effectively inhibited this process. The study demonstrated that supernatants from T helper 17 and γδ-T cells, as well as serum from AS patients, stimulated in vitro bone formation, which was significantly reduced by dual neutralization of IL-17A and IL-17F compared to blocking either cytokine alone. This inhibition was associated with increased expression of the Wnt antagonist DKK1, suggesting a potential mechanism for the observed effects. The findings suggest that targeting both IL-17A and IL-17F could be a promising therapeutic strategy to prevent pathological bone formation [173]. A 48-week phase IIb, randomized, double-blind, placebo-controlled, dose-ranging study that involved 303 patients randomized to receive varying doses of Bimekizumab or placebo every 4 weeks for 12 weeks, followed by re-randomization for a continued treatment period up to 48 weeks, was conducted on patients with Spondylo-Arthritis. The primary endpoint was the Assessment of Spondylo-Arthritis International Society 40 (ASAS40) response at week 12. Results showed significantly higher ASAS40 response rates in Bimekizumab-treated patients compared to placebo, with rapid and sustained improvements in key outcomes such as disease activity, functional status, and quality of life [174]. A preclinical study on bone healing demonstrates that the anti-IL-17 antibody promotes new bone regeneration by enhancing the activity of transcription factors FOXO1 and ATF4, which reduce oxidative stress at the injury site. The treatment was compared with other osteoporotic therapies, including anti-RANKL antibody, alendronate (ALN), and parathyroid hormone (PTH). Micro-CT analysis and histological studies showed that anti-IL-17 significantly improved bone healing, increasing trabecular bone volume, number, and thickness, comparable to PTH and superior to anti-RANKL and ALN. The therapy also elevated the expression of osteogenic markers like Runx-2, OCN, ALP, and type I collagen and boosted bone mineral density and bone strength [175].

#### 4.2.4. ANV419

Is a novel antibody-cytokine fusion protein. It is a potent and highly selective IL-2Rβγ binding agonist, consisting of an antibody specific for the IL-2Rα-binding domain of IL-2. It is designed to stimulate immune cells selectively, enhancing their tumor-killing capabilities while minimizing the activation of immunosuppressive cells. In a phase I/II study ANV419 was administered intravenously every two weeks across six dosage cohorts (3 to 108 mcg/kg). Among thirteen patients with various cancers, ANV419 was tolerated well with only Grade 1 or 2 adverse events, primarily chills and low-grade fever, resolved with antipyretics. Pharmacodynamic assessments revealed a dose-dependent increase in Ki-67 positive CD8 T cells and NK cells, with minimal Treg proliferation up to 48 mcg/kg. Pharmacokinetic data showed a dose-proportional increase in ANV419 plasma concentration with a half-life of 17.6 h at 24 mcg/kg. Four patients continue treatment, with four of eleven patients achieving stable disease. ANV419 demonstrates selective immune activation, particularly enhancing CD8 T cells and NK cells, indicating its potential therapeutic benefit in cancer treatment [176].

The Phase 1 study of ANV419 in patients with relapsed/refractory advanced solid tumors aimed to evaluate the safety, tolerability, maximum tolerated dose (MTD), and recommended phase 2 dose (RP2D) of the drug. Forty patients were enrolled, and the study found the MTD and RP2D to be 243 µg/kg. The treatment demonstrated antitumor activity, evidenced by disease stabilization in many patients and a confirmed partial response in one. These interim findings suggest that ANV419, administered at the established RP2D, offers a promising therapeutic profile for heavily pretreated patients with advanced solid tumors [177].

#### 4.2.5. Canakinumab

Several IL-1β inhibitors have received approval from the FDA for various rheumatological and autoimmune disorders, such as Anakinra, Canakinumab, and Rilonacept [178,179]. The potential of IL1β inhibition using Anakinra or Canakinumab is being investigated as a treatment approach in a diverse array of solid tumor malignancies, whether as a standalone therapy or in conjunction with chemotherapy, immunotherapy, and targeted therapies. This IL-1β inhibitor has shown promise in preclinical models by reducing inflammation and slowing the progression of bone metastases [180]. Studies suggest it could be beneficial in managing bone metastases by targeting the IL-1 signaling pathway [6]. In a preclinical study, IL1B produced by tumor cells drives the metastasis and growth of breast cancer in the bone microenvironment. The presence of IL1B in tumor cells correlated with a higher risk of relapse in both bone and other distant sites. The results demonstrated that IL1B promotes epithelial-to-mesenchymal transition (EMT), invasion, migration, and colonization of bone. Pharmacological inhibition of IL1B using agents like canakinumab and Anakinra significantly reduced metastasis, suggesting potential therapeutic benefits [118]. In another study, Anakinra, or the IL1β antibody, Canakinumab, reduces metastases and nearly eradicates breast cancer growth in the bone [181]. However, these medications promote primary tumor growth. This study focuses on therapeutic drugs that target multiple components of the IL-1 signaling cascade, including caspase-1 inhibitors (e.g., VX765), IL-1β inhibitors (e.g., anakinra), and IL-1 receptor antagonists (e.g., inhibition of IL-1 via MLX01). They found that in vivo, VX765 and Anakinra drastically decreased spontaneous metastasis and metastatic outgrowth in the bone, while MLX01 suppressed primary tumor growth and bone metastasis [181].

#### 4.2.6. Denosumab

Although Denosumab is not an IL-inhibitor but the fact that it is a RANKL ab and this pathway is usually involved with IL function we would like to highlight this drug as a possible combination with other IL inhibitors. Denosumab, which is an entirely humanized monoclonal neutralizing antibody, functions by inhibiting the activation of the RANK/RANKL/OPG signaling pathway via a competitive interaction with RANKL, consequently leading to the suppression of bone resorption caused by osteoclasts [182,183]. The role of Denosumab as a therapeutic agent for various metabolic bone disorders such as postmenopausal osteoporosis, male osteoporosis, and glucocorticoid-induced osteoporosis within the realm of clinical treatment is underscored by its ability to prevent bone loss [184]. Since its inception, Denosumab has exhibited a broad spectrum of effects, unveiling a multifaceted pharmacological profile with substantial promise in addressing diverse clinical conditions like osteoarthritis, bone malignancies, and autoimmune ailments [185,186,187]. Currently, Denosumab is gaining recognition as a feasible treatment choice for individuals with bone metastases originating from malignancies, manifesting both direct and indirect antineoplastic characteristics in both experimental and clinical contexts. In a study that evaluated the relationship between Denosumab use and breast cancer risk in postmenopausal women who were treated previously with bisphosphonates, it reported that the utilization of Denosumab was linked to a modest yet statistically significant 13% reduction in the likelihood of developing breast cancer later on. Over a period of 5 years of monitoring, the overall occurrence of breast cancer was notably lower among those who used Denosumab compared to those who did not [188,189]. Nevertheless, in spite of its innovative attributes, there remains an insufficiency in the clinical application of Denosumab for bone metastasis in malignant tumors, necessitating further investigation into its mechanism of operation [190]. Clinical trials have shown its superiority over bisphosphonates like zoledronic acid in delaying skeletal-related events in patients with bone metastases from breast and prostate cancer [191,192]. A randomized, double-blind clinical study of Denosumab versus zoledronic acid in the treatment of bone metastases in patients with advanced cancer (excluding breast and prostate cancer) or multiple myeloma was conducted to compare and contrast the effectiveness of Denosumab versus Zoledronic acid in terms of reducing the occurrence of skeletal-related events (SREs) among individuals suffering from bone metastases. The findings of this trial suggested that Denosumab exhibited superior performance in terms of delaying the onset of SREs, demonstrating a lower incidence of such events as compared to Zoledronic acid. It was noted that both pharmaceutical agents displayed similar safety profiles; nevertheless, Denosumab emerged as the more favorable option in terms of its overall efficacy in managing bone metastases [192].

An International, double-blind, double-dummy, randomized, active-controlled, phase 3 trial was conducted on patients with newly diagnosed multiple myeloma to compare the efficacy and safety of Denosumab and zoledronic acid in preventing skeletal-related events. Denosumab was non-inferior to zoledronic acid for the time to the first skeletal-related event, with no significant difference in overall survival and similar progression-free survival. Suggesting Denosumab offers an effective alternative to zoledronic acid for patients with multiple myeloma [193]. An international, multicenter, randomized, placebo-controlled, phase 3 trial aimed to assess the efficacy of Denosumab in improving bone metastasis-free survival in women with high-risk early-stage breast cancer. The primary endpoint, bone metastasis-free survival, was not significantly different between the Denosumab and placebo groups, and no significant improvements were observed in key secondary endpoints such as disease-free survival or overall survival. Despite strong preclinical evidence, the study concludes that adjuvant Denosumab did not improve disease-related outcomes in women with early-stage, high-risk breast cancer. The findings suggest that while Denosumab has established benefits for skeletal health, it does not significantly impact bone metastasis-free survival or disease-free survival in this patient population [194]. A study included 50 Chinese patients with breast cancer who received Denosumab treatment. Results showed that 24% of patients experienced skeletal-related events (SREs) within one year of treatment, with a higher incidence in patients with five or more metastatic bone lesions. The median time to the first on-study SRE was not reached. Common adverse events included hypocalcemia (68%), periodontitis (28%), and myalgia (14%), with only three grade III/IV adverse events reported. The study concluded that Denosumab is both effective and well-tolerated in this patient population, particularly among those with multiple metastatic bone lesions [195]. A randomized Phase III trial, as detailed in the manuscript, evaluated the efficacy, safety, and pharmacokinetics of QL1206, a biosimilar of Denosumab, compared to Denosumab in patients with bone metastases from solid tumors. The study concluded that the biosimilar QL1206 is as effective and safe as Denosumab for patients with bone metastases from solid tumors, providing a potentially cost-effective treatment option. The results support the clinical interchangeability of QL1206 with Denosumab [196].

Denosumab, recognized for its potent anti-resorptive properties in the treatment of bone metastases, has been associated with potential side effects that may upregulate alternative signaling pathways to promote osteoclast formation. A study found that anti-RANKL antibody (Denosumab) treatment significantly increased serum IL-8 levels in rheumatoid arthritis (RA) patients. In vitro assays demonstrated that anti-RANKL antibody induced IL-8 production from pre-osteoclast-like cells, promoting osteoclast formation even without RANKL activity. This suggests that IL-8 may compensate for RANKL in osteoclastogenesis, contributing to bone erosion. The study also found that FK506 (tacrolimus) could inhibit IL-8 production, indicating its potential as a combination therapy with anti-RANKL antibody to improve treatment outcomes in RA by reducing osteoclastogenesis-promoting IL-8 levels [197]. This observation warrants further investigation and emphasis on the beneficial use of IL inhibitors in combination with other bone agents in the treatment of bone metastases.

### 4.3. Summary of Clinical Trials and Outcomes

Clinical trials have shown that these medicines are effective. Denosumab, for example, has been demonstrated to reduce the frequency of skeletal-related events while also increasing the quality of life in patients with bone metastases. Tocilizumab and canakinumab are also being investigated, with preliminary findings indicating possible advantages in lowering bone pain and tumor progression. The findings of these studies emphasize the importance of targeted interleukin treatments in the treatment of bone metastases [4,191]. These trials seek to provide a complete approach to controlling bone metastases by combining interleukin inhibitors with standard and new cancer medications. The results of these trials are expected to influence future treatment methods and enhance patient outcomes.

### 4.4. Combinational Therapies

Combining interleukin inhibitors with other treatments, such as chemotherapy, radiation, or other targeted medicines, has promised to overcome resistance and improve therapeutic outcomes.

The CANOPY-1 study, a phase III randomized, double-blind trial, investigated the efficacy of adding canakinumab, a human monoclonal anti-IL-1β antibody, to a first-line treatment regimen of pembrolizumab and platinum-based chemotherapy for patients with advanced or metastatic non-small-cell lung cancer (NSCLC) without EGFR or ALK mutations. Despite a median follow-up of 6.5 months and 21.2 months for progression-free survival (PFS) and overall survival (OS), respectively, both primary endpoints showed no significant improvement with canakinumab; both PFS and OS were similar between the two groups. Despite these findings, canakinumab did offer some improvement in terms of delaying the deterioration of lung cancer symptoms such as chest pain, coughing, and dyspnea. Ultimately, the study concluded that adding canakinumab to the treatment regimen did not extend PFS or OS in this patient population [198]. A case report of a 40-year-old male with SAPHO syndrome, (synovitis, acne, pustulosis, hyperostosis, and osteitis, presents with a range of bone and skin issues) exhibited symptoms including jaw and back pain, joint swelling, weight loss, physical decline, and acne-like skin lesions. Elevated inflammatory markers and imaging showing osteolytic bone lesions confirmed the diagnosis. Initial treatment with glucocorticoids and NSAIDs proved ineffective, leading to high-dose dexamethasone and prednisone use. Due to insufficient response, the patient began daily subcutaneous anakinra injections (100 mg) and Denosumab prior to planned mandibular osteosynthesis. After five months, FDG-PET/MR indicated decreased inflammation and bone pain, but only partial improvement in skin lesions, leading to an assessment of partial remission [199]. Suggesting that Denosumab combined with IL-β inhibitor may have positive effects on bone diseases.

A more recent study explores the potential of IL-17 neutralizing antibodies (NIL17) as a sequential treatment after discontinuing parathyroid hormone (PTH) therapy on bone health. The study reveals that the withdrawal of PTH therapy leads to significant bone loss, increased oxidative stress, and elevated osteoclast activity, evidenced by reduced bone mineral density, bone structure, and higher serum CTX-I levels. Sequential NIL17 therapy effectively mitigates these adverse effects, preserving bone mass and strength, reducing osteoclast numbers, and enhancing oxidative stress markers like FOXO1 and ATF4. The findings suggest that NIL17 can serve as a promising replacement therapy post-PTH discontinuation, offering superior osteoprotective benefits and highlighting the need for further clinical studies to optimize osteoporosis treatment regimens [200].

### 4.5. Treatment Challenges and Limitations

Resistance to interleukin-targeted therapies is a significant challenge. Tumor cells and the bone microenvironment can adapt, reducing the effectiveness of these treatments over time. For example, continuous use of Denosumab can lead to rebound osteoclast activity, necessitating the development of combination therapies to sustain efficacy [4,191]. Targeting interleukins can result in side effects such as infections, liver enzyme elevations, and osteonecrosis of the jaw (ONJ) associated with Denosumab use (Table 2). These adverse effects limit the long-term use of these therapies and require careful management to balance therapeutic benefits with patient safety [4,191]. Long-term efficacy remains a concern, as bone metastases often require prolonged treatment. The chronic nature of bone metastases necessitates sustained therapeutic strategies, and the long-term impacts of interleukin-targeted treatments are still being studied. There is a need for more comprehensive data on the durability of these treatments and their long-term safety profiles [4,191].

### 4.6. Cost Effectiveness

The cost and availability of several therapeutic drugs vary significantly. Tocilizumab (Actemra) is priced at approximately $1092 per 200 mg vial, with costs fluctuating based on dosage and patient weight [201]. This drug is globally available, although insurance coverage varies. Siltuximab (Sylvant) costs approximately $1590 for a 100 mg vial and $6333 for a 400 mg vial, with typical administration being 11 mg/kg every three weeks [202]. Its availability is primarily in major healthcare markets, but its high cost can be a limiting factor. Bimekizumab’s cost is not explicitly available but is estimated to range from $1000 to $10,000 per month, with expanding availability as it gains more approvals. ANV419 is currently in clinical trials, with its post-approval cost yet to be determined. Canakinumab (Ilaris) costs approximately $16,000 per dose, with administration every 4 to 8 weeks, and is available in major markets, although insurance coverage varies. Lastly, Denosumab (Xgeva) is priced at approximately $1800 per injection, typically administered once every 4 weeks, and is widely available, often covered by insurance for approved uses.

## 5. Interleukins as Diagnostic and Prognostic Biomarkers

Interleukins have been identified as crucial diagnostic and prognostic markers in various types of cancer. Studies have highlighted several interleukins with significant implications for cancer diagnosis and prognosis [36,203]. Interleukins have significant potential to improve patient outcomes in the detection and prognosis of bone metastases, particularly in breast cancer. Given their critical roles in immune regulation, tumor growth, metastasis, and bone remodeling, interleukins, such as IL-6, IL-8, IL-1β, and IL-11, have been useful in predicting the clinical stage and prognosis of patients with breast cancer which has metastasized to the bone [6]. It is crucial to identify "high-risk" patients at an early stage in order to put therapies in place that may stop or postpone the development of bone metastases. The key biomarkers that are commonly used include bone turnover markers, microRNAs (miRNAs), circulating tumor DNA (ctDNA), disseminated tumor cells (DTCs), and circulating tumor cells (CTCs) [204]. These markers have the ability to predict the development of bone metastases, which could help identify and treat people at risk early on (Table 3).

Hao et al. categorized the biomarkers linked to bone metastases in cancer, particularly, breast cancer, are divided into three primary categories: mixed, osteoblastic, and osteolytic [205]. Biomarkers that can be identified by proteins and non-coding RNAs (ncRNAs) by liquid biopsies are included in each class. Osteoblastic biomarkers, such as CD74 and Osteopontin, stimulate the production of new bone, whereas osteolytic biomarkers, such as miR-7 and miR-10b, encourage the degradation of existing bone. Mixed biomarkers, such as CXCR4 and RASSF1A, demonstrate both functions [205]. Also, they provide an extensive list of biomarkers used for diagnosing various cancer types. This categorization facilitates targeted therapy by helping to diagnose and prognosticate bone metastases.

In a recent study conducted on prostate cancer (PCa) tissue from humans, IL-38 expression was significantly higher in PCa tissues compared to adjacent non-cancerous tissues, with higher levels associated with a greater proliferation index of tumor cells. The diagnostic value of IL-38 was underscored by its high specificity and sensitivity for detecting PCa, demonstrated by an area under the curve (AUC) of 0.76 in ROC curve analysis. IL-38 expression inversely correlated with the levels of CD8 and PD-1, both important immune markers. Survival analysis revealed that higher IL-38 expression was linked to significantly lower overall survival rates in PCa patients, particularly in those with high IL-38 and low CD8 expression [206]. The role of interleukin in various other types of cancer has been recognized. In hepatocellular carcinoma (HCC) patients with HBV history, interleukin-25 (IL-25) is identified as a potential biomarker for lung metastasis. Elevated IL-25 levels were found to be associated with disease progression and poorer progression-free survival, indicating its prognostic value in HBV-associated HCC [207]. In lower-grade gliomas (LGG), it was demonstrated that IL-6 is a significant prognostic biomarker, with higher expression levels correlating with poorer overall survival (OS). The study identified 146 differentially expressed genes (DEGs) between deceased and surviving patients, with IL-6 being the only immune-related gene significantly associated with prognosis. IL-6’s prognostic value was validated using Kaplan-Meier, restricted cubic spline (RCS), and receiver operating characteristic (ROC) analyses, which confirmed its linear relationship with survival [208]. In oral squamous cell carcinoma (OSCC) it was found that IL1RA expression is significantly reduced in OSCC tissues compared to normal tissues, with expression levels decreasing progressively from hyperplasia to invasive carcinoma. Low IL1RA expression was associated with poor prognosis, including worse disease-free survival (DFS) and overall survival (OS), and correlated with clinicopathological markers such as higher infiltration rates, recurrence, and lymph node metastasis [209]. A study involving 68 Brazilian women with breast cancer examined IL-33 expression across various subtypes and stages of the disease using quantitative polymerase chain reaction (qPCR). The results revealed a significant upregulation of IL-33 in breast cancer tissues, particularly in the Triple-negative and Luminal B subtypes, compared to healthy controls. Notably, Luminal B patients exhibited higher IL-33 levels than Luminal A patients, with the highest expression in advanced TNM stage IV cases. Furthermore, chemotherapy-naïve patients had elevated IL-33 expression, which decreased following neoadjuvant chemotherapy, suggesting that chemotherapy might reduce tumor aggressiveness by suppressing IL-33. These findings indicate that IL-33 could serve as a prognostic marker [210]. In patients with unresectable or metastatic renal cell carcinoma (RCC) treated with immune checkpoint inhibitors (ICIs), IL-1β and IL-6 were both found to significantly influence progression-free survival (PFS) and overall survival (OS). IL-1β, known for inducing tumor angiogenesis and promoting immune evasion, was associated with worse outcomes, reinforcing its role as a negative prognostic factor. Similarly, IL-6, a cytokine involved in inflammation and immune responses, demonstrated a strong correlation with poor prognosis. Elevated levels of IL-6 were significantly associated with reduced OS, suggesting that IL-6 might be a critical biomarker for patient stratification and treatment optimization [211].

In primary breast cancer tumors, IL-11 was found to be expressed in 17% of the primary breast tumors examined, with a significant association between IL-11 expression and low tumor grade (grades 1–2) (*p* = 0.05). Tumors expressing IL-11 mRNA had a statistically significant higher rate of bone metastases (80%) compared to IL-11 negative tumors (37%) (*p* = 0.002). This study demonstrates IL-11 mRNA presence in primary invasive breast tumors, suggesting that IL-11 could serve as a biological predictive factor for bone metastases [212]. Interleukin-3 (IL3) was identified as a promising prognostic marker for bone metastases. IL3 was part of a 15-gene panel consistently overexpressed in bone metastases across various cancer types. Validation using exosomal mRNA from patient samples revealed significant upregulation of IL3 in both breast and lung cancer patients with bone metastases compared to those without. ROC curve analysis demonstrated high sensitivity and specificity for IL3 in distinguishing between primary cancers with and without bone metastases, sometimes reaching 100% [213]. Elevated levels of IL-17 and its signaling components in the bone microenvironment are associated with poor prognosis in patients with bone metastases. This association suggests that IL-17 could be used as a biomarker to predict disease progression and response to therapy in metastatic cancer patients.

By utilizing biomarkers like these interleukins, clinicians can steer therapeutic choices, leading to particular interventions that target the distinctive molecular pathways fueling bone metastases. Furthermore, observing variations in interleukin levels throughout treatment, such as monitoring adjustments in IL-8 or IL-11 post-treatment [213], can furnish a valuable understanding of treatment responses and support the enhancement of patient outcomes. The use of personalized medicine techniques driven by interleukin biomarkers has the potential to revolutionize the treatment of bone metastases through tailored and effective interventions that address the particular biological processes driving the disease’s advancement.

Grasping the complex interplay among tumor cells, bone cells, and interleukins is imperative for formulating efficient diagnostic and prognostic methodologies. A focal point on interleukin signaling pathways, particularly IL-11, can pave the way for fresh therapeutic pathways to prevent and address breast cancer bone metastasis [52]. Additionally, delving into the fluctuating patterns of inflammatory markers following radiotherapy for painful bone metastases could yield precious insights into treatment responses, fostering personalized approaches to enhance pain alleviation and clinical outcomes [213]. This all-encompassing strategy of capitalizing on interleukins as diagnostic indicators and therapeutic targets highlights the scope for personalized and efficient bone metastases management in breast cancer.

## 6. Gaps in Current Research and Future Directions

### 6.1. Research Gaps

Examining the gaps in the literature concerning interleukins and bone metastases requires a complex understanding of the current limitations and the areas that require further investigation. Numerous studies have highlighted the critical importance of interleukins, particularly IL-1, IL-6, and IL-8, in promoting bone metastases from breast cancer through different signaling pathways. While preclinical research provides evidence for their potential contributions to neoplastic progression and osseous deterioration, clinical research focuses mostly on IL-2 and IL-12, which may have latent anti-metastatic effects. However, the lack of conclusive results highlights the need for comprehensive research to figure out the complex cellular and molecular mechanisms behind interleukins in bone metastases. Combining these observations could provide valuable information about treatment strategies aimed at blocking interleukin-mediated intercellular communication between neoplastic and osseous factions. Furthermore, the potential impact of anti-inflammatory interleukins like IL-4 and IL-10 has not received sufficient attention in the sphere of bone metastases, despite indications of their ability to regulate immune reactions and osteoclast behavior. Research focusing on the specific mechanisms by which interleukins influence these interactions, particularly their effects on bone remodeling processes, may provide important new information on possible targets and approaches for treatment [7]. Understanding how interleukins shape the immunological milieu within bone, including their effects on other immune cells, implications for tumor immunity, and immune evasion, is critical for developing tailored therapies to effectively fight bone metastases [52].

### 6.2. Future Directions

Greten and Grivennikov conducted a study that sheds insight on the critical role that inflammation plays in cancer progression and metastasis, emphasizing the immune system’s dualistic character in tumor regulation [7]. The same authors emphasize the role of chronic inflammation in tumor progression, emphasizing the importance of understanding and targeting inflammatory processes in order to develop more effective cancer therapy techniques. Furthermore, future research efforts could focus on identifying novel therapeutic targets within the realm of ILs to disrupt the promotion of metastatic signals and hamper the ongoing progression of bone metastases [7]. These proposed inquiries have the potential to clarify pioneering treatment methodologies aimed at the IL-driven pathways orchestrating the dissemination of metastatic cells to the bone microenvironment [6]. Further research is needed to elucidate the precise molecular mechanisms by which interleukins influence the bone metastatic niche. This includes detailed studies on the signaling pathways involved and their interactions with other cytokines and cellular components. Validation of interleukins as diagnostic and prognostic biomarkers. This will aid in patient stratification, allowing for personalized treatment approaches and better prediction of disease progression and treatment response. The synergistic effects of combining interleukin-targeted therapies with existing treatments such as chemotherapy, radiotherapy, and other targeted agents. This approach may help overcome resistance and improve therapeutic outcomes. Comprehensive clinical trials are needed to evaluate the long-term efficacy and safety of interleukin-targeted therapies. These trials should focus on different cancer types and stages to assess the broad applicability of these treatments.

## 7. Conclusions

The role of interleukins in the pathogenesis and progression of bone metastases is multifaceted and significant. Pro-inflammatory interleukins such as IL-1, IL-6, and IL-8 facilitate osteoclastogenesis, tumor proliferation, and angiogenesis, thereby contributing to the complex interactions within the bone microenvironment that drive metastatic disease. Conversely, anti-inflammatory interleukins like IL-4, IL-10, and IL-13 exhibit potential protective effects by modulating immune responses and inhibiting osteoclast activity, offering promising avenues for therapeutic intervention.

The intricate signaling pathways involving NF-κB, JAK/STAT, and MAPK are crucial mediators of the effects of these interleukins, influencing tumor cell survival, immune cell recruitment, and bone remodeling processes. Understanding these pathways provides valuable insights into potential therapeutic targets for disrupting the progression of bone metastases.

Current therapeutic strategies, including the use of Denosumab, Tocilizumab, and emerging agents such as Bimekizumab and ANV419, demonstrate the potential of targeting interleukin-mediated pathways. However, challenges such as therapeutic resistance, adverse effects, and the need for sustained efficacy highlight the complexities of treating bone metastases. The diagnostic and prognostic potential of interleukins further underscores their importance in managing bone metastases. Biomarkers like IL-6, IL-8, IL-1β, and IL-11 offer opportunities for early detection, patient stratification, and personalized treatment approaches, enhancing the clinical management of metastatic disease.

The dualistic nature of interleukins underscores the complexity of their roles in bone metastases. Understanding these opposing effects is crucial for developing targeted therapies that can disrupt the pathological processes and enhance clinical outcomes for patients with bone metastases.

## Figures and Tables

**Table 1:** Therapeutic Agents Targeting Interleukins.

Therapeutic Agent	Target Interleukin	Mechanism of Action	Clinical Application	Challenges/Limitations
Tocilizumab	IL-6	Inhibits IL-6 receptor	Rheumatoid arthritis, potential in reducing bone metastases	Infections, liver enzyme elevation
Siltuximab	IL-6	Neutralizes IL-6 bioactivity	Prostate cancer, ovarian cancer	Resistance, cytokine release syndrome
Bimekizumab	IL-17A and IL-17F	Dual inhibition of IL-17A and IL-17F	Psoriatic arthritis, potential in bone healing	Long-term safety, high cost
ANV419	IL-2Rβγ	Selective IL-2Rβγ binding agonist	Advanced solid tumors	Grade 1/2 adverse events
Canakinumab	IL-1β	IL-1β inhibition	Rheumatoid arthritis, potential in bone metastases	Expensive, risk of infections
Denosumab	RANKL	Inhibits RANK/RANKL interaction	Bone metastases from solid tumors	Osteonecrosis of the jaw, hypocalcemia

**Table 2:** This table provides a comprehensive overview of drugs targeting interleukins, including their doses, clinical effects, side effects, and findings from clinical and animal studies. Each drug has demonstrated effectiveness in reducing inflammation and improving clinical outcomes in various conditions. However, side effects such as infections and elevated liver enzymes are common, necessitating careful monitoring. Clinical and animal studies provide valuable insights into the mechanisms of action and potential therapeutic benefits of these drugs, guiding their use in clinical practice and future research directions.

Drug	Dose	Clinical Effect	Side Effects	Clinical Study	Animal Study
Tocilizumab	8 mg/kg IV every 4 weeks	Reduces inflammation, improves symptoms in RA and other conditions	Infections, elevated liver enzymes	Effective in reducing bone metastases in preclinical studies; used in RA and Castleman’s disease; investigated in other conditions (Crohn’s disease, SLE)	Reduced bone metastases in animal models of breast cancer; inhibited cell survival and reduced expressions of Stat3, VEGF, and RANK in MDA-231 cells
Siltuximab	11 mg/kg IV every 3 weeks	Neutralizes IL-6, reduces tumor cell survival, enhances chemotherapy effects	Infections, neutropenia, thrombocytopenia	Inhibits proliferation of prostate cancer cells in vitro; extends disease stability; reduces levels of TNF-α, IL-1, CCL2, CXCL12, and VEGF	Inhibits androgen-dependent prostate cancer progression in mice; reduces cachexia levels in prostate cancer models
Bimekizumab	160–320 mg SC every 4 weeks	Dual neutralization of IL-17A and IL-17F, improves bone health	Infections, nasopharyngitis, oral candidiasis	Effective in hidradenitis suppurativa and spondyloarthritis; improves key outcomes such as disease activity, functional status, and quality of life	Inhibits osteogenic differentiation and bone formation in human periosteum-derived cell models; improves bone healing and regeneration in osteoporotic models
ANV419	243 µg/kg IV every 2 weeks	Enhances tumor-killing capabilities of immune cells, minimizes immunosuppressive cell activation	Chills, low-grade fever	Phase I/II study showed well-tolerated, disease stabilization in solid tumors; phase I study demonstrated antitumor activity with stable disease and partial response in advanced tumors	Stimulates immune cells selectively, enhancing tumor-killing capabilities while minimizing activation of immunosuppressive cells
Canakinumab	150–300 mg SC every 4 weeks	Reduces inflammation, slows bone metastasis progression	Infections, neutropenia, thrombocytopenia	Ongoing trials for various solid tumor malignancies; reduces metastasis and bone tumor growth in preclinical studies	Inhibits breast cancer growth and bone metastasis in preclinical models; reduces metastasis and metastatic outgrowth in bone with VX765 and Anakinra
Denosumab	120 mg SC every 4 weeks	Inhibits bone resorption, delays skeletal-related events, improves quality of life	Hypocalcemia, osteonecrosis of the jaw	Effective in delaying skeletal-related events in breast and prostate cancer; superior to bisphosphonates in delaying skeletal-related events	Increases serum IL-8 levels, promoting osteoclast formation even without RANKL activity; combination with IL inhibitors may enhance treatment outcomes

**Table 3:** This table provides an overview of the interleukins involved in bone metastases, highlighting their roles as diagnostic and prognostic biomarkers.

Interleukin	Type	Role	Diagnostic Biomarker	Prognostic Biomarker
IL-1	Pro-inflammatory	Promotes osteoclastogenesis, tumor proliferation, angiogenesis	Elevated IL-1 levels in patient serum indicate active inflammation and metastasis	High IL-1 levels correlate with increased tumor burden and poor prognosis
IL-6	Pro-inflammatory	Promotes osteoclastogenesis, tumor proliferation, angiogenesis	Elevated IL-6 levels indicate tumor activity and systemic inflammation	High IL-6 levels correlate with advanced disease and poor prognosis
IL-8	Pro-inflammatory	Stimulates osteoclastogenesis, promotes angiogenesis	Elevated IL-8 levels indicate active metastatic process	High IL-8 levels correlate with increased metastatic potential and poor prognosis
IL-11	Pro-inflammatory	Promotes osteoclastogenesis and bone degradation	Elevated IL-11 levels are associated with active bone resorption	High IL-11 levels correlate with bone metastasis and worse clinical outcomes
IL-17	Pro-inflammatory	Enhances osteoclastogenesis, promotes tumor cell survival	Elevated IL-17 levels indicate aggressive disease	High IL-17 levels correlate with poor survival and increased bone destruction
IL-18	Pro-inflammatory	Modulates tumor microenvironment and tumor development	Elevated IL-18 levels are associated with tumor progression	High IL-18 levels correlate with poor patient outcomes
IL-4	Anti-inflammatory	Inhibits osteoclast activity, modulates immune responses	Elevated IL-4 levels indicate anti-inflammatory response	High IL-4 levels correlate with better clinical outcomes and reduced bone resorption
IL-10	Anti-inflammatory	Inhibits osteoclast activity, promotes immune regulation	Elevated IL-10 levels indicate anti-inflammatory state	High IL-10 levels correlate with reduced tumor progression and better prognosis
IL-13	Anti-inflammatory	Inhibits osteoclast activity, modulates immune responses	Elevated IL-13 levels indicate anti-inflammatory environment	High IL-13 levels correlate with better prognosis and reduced metastatic potential

Elevated levels of pro-inflammatory interleukins (IL-1, IL-6, IL-8, IL-11, IL-17, and IL-18) generally indicate active disease and poor prognosis, whereas elevated levels of anti-inflammatory interleukins (IL-4, IL-10, and IL-13) are associated with better clinical outcomes and reduced metastatic potential. This information is crucial for developing targeted therapies and improving patient stratification and personalized treatment approaches.
